# Reasoning about climate change

**DOI:** 10.1093/pnasnexus/pgad100

**Published:** 2023-05-02

**Authors:** Bence Bago, David G Rand, Gordon Pennycook

**Affiliations:** Institute for Advanced Study in Toulouse, University of Toulouse 1—Capitole, 1 esplanade de l’Université, Toulouse 31080, France; Artificial and Natural Intelligence Toulouse Institute, Federal University of Toulouse Midi-Pyrénées, Toulouse 31000, France; Sloan School, Massachusetts Institute of Technology, Cambridge, MA 02139, USA; Department of Brain and Cognitive Sciences, Massachusetts Institute of Technology, Cambridge, MA 02139, USA; Hill/Levene Schools of Business, University of Regina, Regina, SK S4S 0A2, Canada

## Abstract

Why is disbelief in anthropogenic climate change common despite broad scientific consensus to the contrary? A widely held explanation involves politically motivated (system 2) reasoning: Rather than helping uncover the truth, people use their reasoning abilities to protect their partisan identities and reject beliefs that threaten those identities. Despite the popularity of this account, the evidence supporting it (i) does not account for the fact that partisanship is confounded with prior beliefs about the world and (ii) is entirely correlational with respect to the effect of reasoning. Here, we address these shortcomings by (i) measuring prior beliefs and (ii) experimentally manipulating participants’ extent of reasoning using cognitive load and time pressure while they evaluate arguments for or against anthropogenic global warming. The results provide no support for the politically motivated system 2 reasoning account over other accounts: Engaging in more reasoning led people to have greater coherence between judgments and their prior beliefs about climate change—a process that can be consistent with rational (unbiased) Bayesian reasoning—and did not exacerbate the impact of partisanship once prior beliefs are accounted for.

**Significance Statement**

It is commonly argued that reasoning exacerbates political bias via identity-protective cognition. This theoretical account has had a particular influence on the explanation of partisan differences in the context of global warming. According to this account, people exert mental effort to defend their political identities by disputing identity-inconsistent information. However, our results provide no support for this account over other accounts. Beyond raising theoretical questions about how people reason about climate change, our findings suggest a potential alternative pathway for addressing it. Instead of focusing on interventions that try to decrease partisanship saliency when communicating about science, interventions aimed at providing accurate information about climate change may be effective in the long run.

## Introduction

Skepticism about climate change and its human origins represents a major impediment to the adoption of climate change mitigation policies (1–3). One of the most commonly cited reasons for climate change denial is political partisanship or ideologies (4). In the United States, for example, people on the political right are more likely to believe that climate change is a hoax or that it is not caused by human activities (2, 5–8). What is more, people with greater numerical ability and cognitive sophistication show *more pronounced* partisan differences in climate change beliefs, rather than greater agreement with the scientific consensus (9–13). That is, having stronger cognitive ability appears to not protect against climate misperceptions but instead bolster views that align with one's political identity.

The most popular explanation of this result is provided by the motivated system 2 reasoning (MS2R) framework (11, 14–16). Motivated reasoning has been used in connection with a number of processes and motivations, but in this research, we specifically focus on *political* motivations, as they have been argued to be the primary drivers of climate change disbelief (11). This MS2R framework can be interpreted from the point of view of the dual-process perspective (17–19), which distinguishes between two types of reasoning processes: intuition (system 1) and deliberation (system 2). While intuition is considered a low-effort, quick, automatic response to stimuli, deliberation is a more effortful, time-consuming process. The MS2R framework asserts that cognitive abilities are linked to greater polarization because deliberation facilitates politically motivated reasoning: When faced with new evidence, engaging in deliberation better allows one to discredit the evidence if it is not congenial to one's identity and partisan commitments (and vice versa when it is congenial). As a result, there are large partisan differences in what evidence is deemed credible, eventually leading to substantial polarization in beliefs. In the language of dual-process theory, deliberative reasoning processes are triggered to rationalize or justify identity-consistent intuitive impulses. In the context of climate change, this would mean that deliberation leads Republicans to reject evidence in favor of climate change (to protect their partisan identity), while deliberation leads Democrats to reject evidence questioning climate change (10, 11, 20–23). If more cognitively sophisticated people engage in more deliberation, they will be better at aligning their judgments of evidence about climate change with their respective political identities.

This theory has enormous practical importance because, if it is true, common strategies such as educating people or making them more reflective will not be effective against climate change denial. In fact, such strategies will only serve to *increase* partisan differences (10, 23, 24) (although there is evidence questioning this assumption (25–27)). Furthermore, from a theoretical perspective, this “MS2R” account stands in stark contrast to a common dual-process perspective—the “classical reasoning” view—whereby system 2 reasoning is thought to typically facilitate accuracy in a variety of decision-making tasks (18, 28, 29). Put differently, the classical reasoning account posits that when people engage in deliberation, they tend to form more accurate beliefs, regardless of the partisan or identity alignment of the propositions that they are deliberating about (29, 30).

However, there are two serious limitations of the prior empirical research in this area. First, political identity is correlated with—but meaningfully separable from—people's prior beliefs about climate change (31). In particular, Democrats are much more likely to believe that climate change is caused by human activity than Republicans. Yet, many Republicans do believe in anthropogenic climate change, and some Democrats do not, meaning that partisanship and priors are meaningfully distinct constructs. For example, a recent Pew survey found that 53% of conservative Republicans believe that human activity contributes to global warming to at least some degree, while 8% of moderate Democrats think that it does not (5). Yet most studies claiming to provide evidence of politically motivated reasoning have not measured these prior beliefs, which is highly problematic for making strong claims about politically motivated reasoning (31–33). Although partisanship might influence prior beliefs, many other factors also contribute to beliefs, such as who people judge trustworthy as well as family environment or life experiences (12), and prior beliefs may also influence partisanship. Thus, effects driven by prior beliefs do not provide positive evidence in support of politically motivated reasoning.

Indeed, recent correlational work finds that controlling for prior beliefs related to climate change nullifies the correlation between cognitive sophistication and partisan bias; instead, higher cognitive reflection was associated with placing greater emphasis on prior beliefs when evaluating new information (31). While evaluating new evidence in light of prior beliefs is sometimes called “confirmation bias” and can be a vehicle for politically motivated reasoning in so much as political identities influence prior beliefs, it is also possible that such evaluation can be entirely rational and unbiased from a Bayesian perspective^1^ when there is uncertainty about the reliability of sources (34–38). When considering evidence that is inconsistent with your prior beliefs, it can be rational to conclude that it is more likely that the information source is unreliable than it would be to take the stance that everything that (or much of what) you know about a topic is wrong. It is therefore essential to account for prior beliefs when attempting to test for politically motivated reasoning. Any relationships with identity that are not robust to controlling for prior beliefs do not provide *positive* evidence for politically motivated reasoning because they can be consistent with *either* political *or* accuracy motivations. Indeed, distinguishing the effects of prior beliefs and partisanship is important and common in the literature, even among proponents of the MS2R account, best described by Kahan (39): “Under [motivated system 2 reasoning], the signature feature of this form of information processing is the opportunistic adjustment of the weight-assigned evidence conditional on its conformity to positions associated with membership in identity-defining affinity groups. In Bayesian terms, there is an endogenous relationship between the likelihood ratio and a person's political predispositions. It is this entanglement that distinguishes politically motivated reasoning from a normative conception of Bayesian information processing, in which the weight (likelihood ratio assigned) evidence is determined on the basis of valid, truth-seeking criteria independent of an individual's cultural identity. [Motivated system 2 reasoning] also distinguishes politically motivated reasoning from cognitively biased forms of information processing in which the likelihood ratio is endogenous to some non-truth-seeking influence other than identity protection, such as an individuals’ priors in the case of confirmation bias,” although the effects of prior beliefs and partisanship have not been sufficiently empirically investigated in the context of investigating the apparent role of deliberation (39, 40).

Second, past research on MS2R has relied upon correlating individual differences in cognitive sophistication (e.g. cognitive reflection, numeracy, and education) with the extent of partisan differences on politicized issues (9, 11, 41). Although it is generally thought that people scoring higher on cognitive sophistication scales are better at deliberation than people scoring lower on these scales, they also tend to differ in many other aspects. For example, they tend to generate different intuitions on many reasoning tasks (i.e. people who are more cognitively sophisticated also have different prior beliefs and knowledge than those who score lower (42, 43)). Thus, because this approach is correlational, it does not allow for the direct identification of causal effects of deliberation.

### Current research

In the current research, we address both of these limitations. First, we provide a causal test of the role of intuition and deliberation on how people evaluate pro climate change and contra climate change arguments by forcing some participants to make judgments under cognitive load and time pressure. Second, we measure prior beliefs about climate change by asking how serious risk participants believe climate change to be and how much they agree that human activity causes climate change.

This paradigm allows us to shed new light on competing accounts of the role of deliberation in argument evaluation surrounding climate change: Does deliberation magnify partisan bias, consistent with the MS2R framework (11)? Or does it facilitate accurate assessments, consistent with a more classical perspective on reasoning (30, 31, 37)?

Furthermore, we specify a third alternative. Previous research (e.g. studying blatantly false political news posts (30)) has argued that the classical reasoning approach simply predicts that more deliberation will lead to increased objective accuracy, defined here as holding a position more consistent with the scientific consensus on climate change. However, most people do not actually have direct access to the information needed to know the objectively accurate answer, particularly in the context of complicated technical issues like climate change. Thus, the classical reasoning account would not necessarily predict that deliberation leads to more objectively accurate views. Instead, accuracy-motivated deliberation may lead to improved coherence between one's existing directly relevant beliefs and the stimuli being presented. That is, deliberation may increase the extent to which one evaluates whether new information makes sense in light of the relevant beliefs/knowledge that one has developed based on previous information that one has encountered (a process that, as discussed above, can be consistent with unbiased, rational Bayesian updating (34–37)). In this case, deliberation should magnify differences based on prior beliefs. As a result, finding that deliberation increases coherence with prior beliefs could be consistent with either a motivated or rational account.

In our experiments, we asked participants to indicate how much they agreed with politically neutral arguments about climate change (meaning that there were no references in them to specific policies or to politics in any way). These arguments were taken from “procon.org,” a website that collects arguments that were made in real life about several different topics. Arguments were content counter-balanced, such that for each statement, we created a pro and contra version, one of which was randomly assigned to a given participant; participants never saw both the pro and contra versions of the same argument. Altogether, they were presented with six arguments (half contra and half pro). Table 1 shows the pro and contra versions of an example item from our experiment (for a complete set of statements, see Table S1).

**Table 1.** Table shows an example of the argument items that we used in this experiment.

Pro climate change	Contrary to climate change
Average temperatures on Earth have increased at a rate far faster than can be explained by natural climate changes. A 2008 study compared data from tree rings, ice cores, and corals over the past millennium with recent temperature records. The study created the famous “hockey stick” graph, showing that the rise in Earth's temperature over the preceding decade had occurred at a rate faster than any warming period over the last 1,700 years. In 2012, the Berkeley scientists found that the average temperature of the Earth's land increased 2.5°F over 250 years (1750–2000), with 1.5°F of that increase in the last 50 years.Lead researcher Richard A. Muller, PhD, said that “it appears likely that essentially all of this increase (in temperature) results from the human emission of greenhouse gases.” In 2013, a surface temperature study published in *Science* found that global warming over the past 100 years has proceeded at a rate faster than at any time in the past 11,300 years. According to the IPCC's 2014 Synthesis Report, human actions are “extremely likely” (95–100% confidence) to have been the main cause of the 20th century global warming, and the surface temperature warming since the 1950s is “unprecedented over decades to millennia.”	Earth's climate has always warmed and cooled, and the 20th century rise in global temperature is within the bounds of natural temperature fluctuations over the past 3,000 years. Although the planet has warmed 1–1.4°F over the 20th century, it is within the ±5°F range of the past 3,000 years. A 2003 study by researchers at the Harvard–Smithsonian Center for Astrophysics found that “many records reveal that the 20th century is probably not the warmest nor a uniquely extreme climatic period of the last millennium.”A 2005 study published in *Nature* found that “high temperatures—similar to those observed in the 20th century before 1990—occurred around AD 1000 to 1100” in the Northern Hemisphere. A 2013 study published in Boreas found that summer temperatures during the Roman Empire and Medieval periods were “consistently higher” than temperatures during the 20th century. According to a 2010 study in the Chinese Science Bulletin, the recent global warming period of the 20th century is the result of a natural 21-year temperature oscillation and will give way to a “new cool period in the 2030s.”

Since this is an untested way to measure reasoning about climate change, we started with a pilot study that conceptually replicated prior correlational results regarding cognitive sophistication (the extent to which people are able and willing to engage in deliberation, as measured by the Cognitive Reflection Test (CRT) (44)) and belief in climate change (see Supplementary material Section B, Table S2, and Figs. S1–S3). Consistent with prior work (31), we found that (i) cognitive sophistication is associated with politically polarized evaluations of our climate change arguments but (ii) accounting for differences in prior beliefs about climate change nullifies this interaction between cognitive sophistication and political partisanship. Instead, more cognitive sophisticated participants actually condition their judgments on coherence with prior beliefs rather than partisanship per se.

In our main experiments, we then recruited American participants from Lucid (quota matched to the national distribution on age, gender, ethnicity, and geographic region) and experimentally induced intuitive responding for randomly selected participants to provide a *causal* test of the role of intuition versus deliberation on climate argument evaluation. Deliberation is highly dependent on available working memory capacities and tends to take more time (compared with intuitive responses) (17, 45). Hence, to minimize the extent of deliberation, we applied a working memory load and a strict response deadline (28 s)^2^ to participants in the “intuitive response” condition. More concretely, before being presented with any individual argument, participants had to memorize a dot pattern in a grid and keep it in mind while reading the argument and giving an intuitive agreement score under the time deadline. Conversely, participants in the “deliberative response” condition were presented with the arguments without any constraint (and hence were free to deliberate). The goal of this manipulation was therefore not to induce deliberation per se, but to contrast a condition where participants are free to deliberate as they would normally with a condition where deliberation was severely restricted. This provides a more naturalistic (and conservative) test of the role of deliberation (46–48). Participants were randomly assigned to one or the other experimental condition (between subject). For further details, see Materials and methods (including discussion of a second response that was collected in the intuitive condition where participants could make a subsequent deliberative choice).

According to the MS2R account, when people are able to engage in more reasoning in the deliberative response condition, they should show increased agreement with arguments that are concordant to their partisanship. This yields four specific predictions:

- Deliberation will increase agreement with con arguments for Republicans, even when controlling for prior beliefs.
- Deliberation will decrease agreement with pro arguments for Republicans, even when controlling for prior beliefs.
- Deliberation will decrease agreement with con arguments for Democrats, even when controlling for prior beliefs.
- Deliberation will increase agreement with pro arguments for Democrats, even when controlling for prior beliefs.

If deliberation simply facilitates accurate beliefs (defined in this case as beliefs that are more consistent with the scientific consensus), people will increase agreement with pro arguments and decrease agreement with contra arguments after deliberation, regardless of their partisanship or prior beliefs.

Finally, if deliberation facilitates the coherence between one's prior beliefs and evaluation of new information, we should find that deliberation increases agreement with arguments that are consistent with the participant's prior beliefs about climate change while decreasing agreement with arguments that are inconsistent with the participant's prior beliefs. This yields four specific predictions:

- Deliberation will increase agreement with con arguments for climate deniers, even when controlling for partisanship.
- Deliberation will decrease agreement with pro arguments for climate deniers, even when controlling for partisanship.
- Deliberation will decrease agreement with con arguments for climate believers, even when controlling for partisanship.
- Deliberation will increase agreement with pro arguments for climate believers, even when controlling for partisanship.

It is important to remember that these predictions are all focused on the role of *deliberation* and how it interacts with partisanship and prior beliefs. Whether or not there are main effects of partisanship and prior beliefs on argument evaluation (not moderated by deliberation) is a separate question. Indeed, prior evidence indicates that both partisanship and prior beliefs are associated with climate views (31). However, if such relationships are not magnified by deliberation, then, this suggests that they are not driven by reasoning processes per se.

To test these various predictions regarding the effect of deliberation, we used linear mixed-effect models with crossed random effects for participants and items, including the most complex random effect terms that converged. For the specification of random effects used in the various models, see Materials and methods section Table 2. Our models take agreement ratings as the dependent variable, and as independent variables, we include prior belief in climate change, partisanship, argument type (pro or contra), and response type (intuitive or deliberative response condition). The MS2R account predicts a three-way interaction between partisanship, response type, and argument type, while the specific prior belief account predicts a three-way interaction between specific prior beliefs, argument type, and response type. If deliberation simply facilitates accurate beliefs, we should find a two-way interaction between the response type and the argument type (in a model without partisanship or prior beliefs).

**Table 2.** Table shows all the fixed and random effects that were included in the models in both studies. In all models, the intercept was allowed to vary over subjects and item contents.

	Fixed	Random slope over subjects	Random slope over item contents
Study 1	Argument type, condition (0—intuitive, 1—deliberative), partisanship, and belief	Argument type	Belief
	Argument type and condition	Argument type	—
Study 2	Argument type, condition (1—intuitive, 1—deliberative), partisanship, and belief	Argument type	Argument type
	Argument type and condition	Argument type	Argument type

We conducted two studies that allow us to discern the causal effect of deliberation on climate change beliefs. These studies were equivalent except in two aspects. In study 1, participants received both partisanship and prior belief questions at the end, after all the climate arguments items, while in study 2, they received these questions prior to experimental manipulation. Moreover, in study 1, prior belief was assessed using a single question assessing the belief that human activity causes climate change, while in study 2, prior belief was defined using an average of that question and a second question assessing the risk that climate change poses to humanity.

## Results

### Separability of partisanship and prior beliefs about climate change

**Fig. 1.**
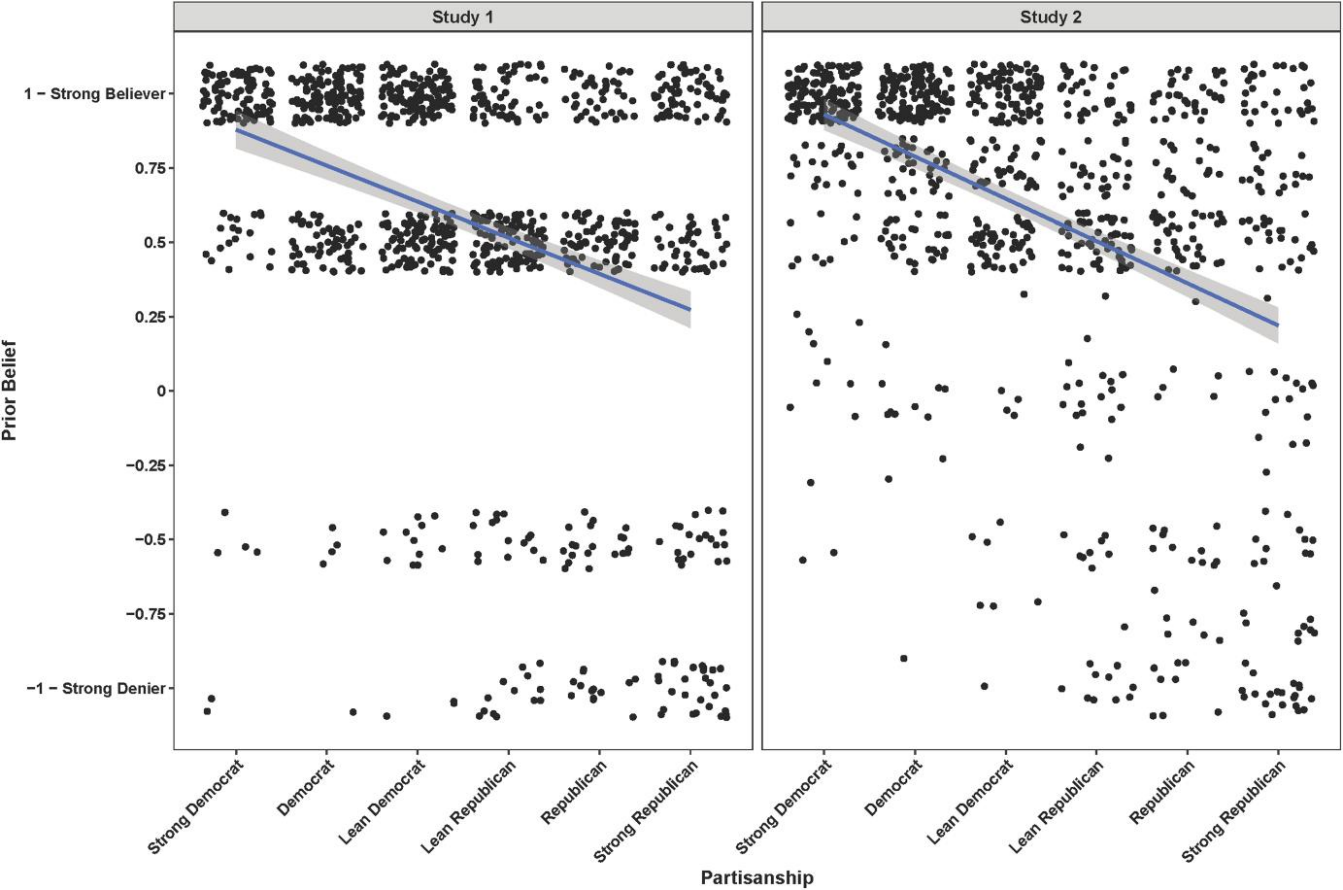
Distribution of specific prior beliefs (i.e. people who believe versus who deny climate change and its consequences) plotted as a function of partisanship in our studies. Although the two measures are moderately correlated, they are meaningfully distinct.

We begin by documenting a motivation for our work—the disconnect between partisanship and prior beliefs. Although there is a moderate correlation between prior beliefs about climate change and partisanship, *r* = 0.34, *P* < 0.0001 (study 1), and *r* = 0.44, *P* < 0.0001 (study 2), it is clear that these measures are meaningfully separable. As shown in Fig. 1, a majority of participants from both parties believed in human-caused climate change. Thus, being Republican does not necessarily mean that one is a climate denier.

### Main experiments

**Fig. 2.**
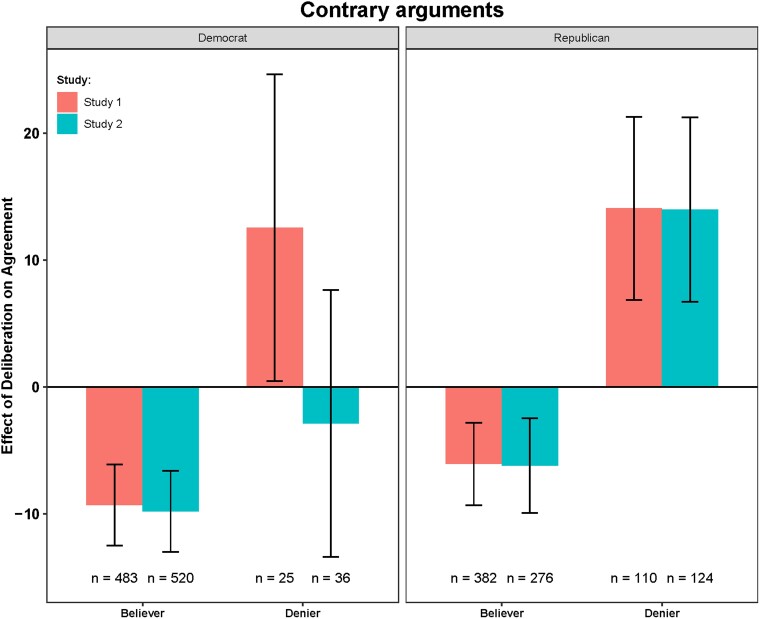
Difference in average agreement scores between intuitive and deliberative ratings as a function of prior belief (believer/denier) and politics (Democrats/Republicans) on contrary to climate change arguments. Bars are mean differences between the agreement scores in the deliberative and the intuitive conditions. Error bars are 95% CI. Negative values indicate that deliberation causes higher agreement scores, and positive values indicate that deliberation causes lower agreement scores.

**Fig. 3.**
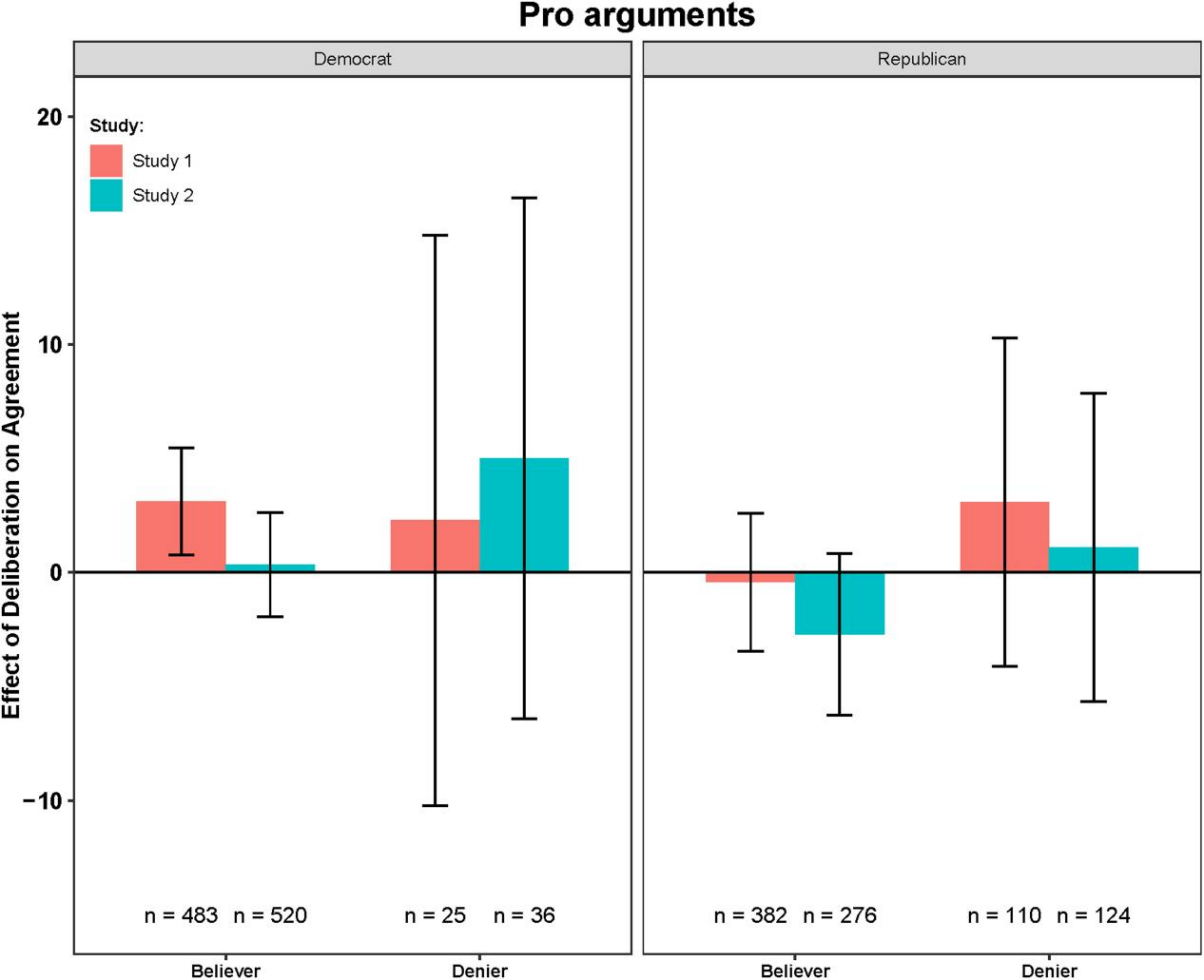
Difference in mean agreement scores between intuitive and deliberative ratings as a function of prior belief (believer/denier) and politics (Democrats/Republicans) on pro climate change arguments. Bars are mean differences between the agreement scores in the deliberative and the intuitive conditions. Error bars are 95% CI. Negative values indicate that deliberation causes higher agreement scores, and positive values indicate that deliberation causes lower agreement scores.

We now turn to our key question of interest and examine the difference in agreement with each statement between participants in the intuitive response condition and the deliberative response condition. This difference in agreement (i.e. the effect of deliberation) for each argument type is shown in Figs. 2 and 3, split by partisanship and prior belief (for graphs of averages and distributions in the different conditions, see Figs. S4–S7).

We see similar results across both studies. Our model includes the argument type, response condition, partisanship, prior beliefs, and relevant interactions. We observed that both partisanship (study 1: *b* = −10.12, *P* = 0.007; study 2: *b* = −5.76, *P* = 0.051) and beliefs (study 1: *b* = −10.12, *P* < 0.0001; study 2: *b* = −9.89, *P* = 0.0002) have a direct effect on argument evaluation (i.e. they significantly interact with the argument type), such that Republicans and climate deniers are less likely to agree with pro than with contra arguments and vice versa for Democrats and climate believers. However, we are primarily interested in whether deliberation *amplifies* any of these effects. If deliberation amplifies the effect of partisanship that we observed above, this would provide unique positive support for the MS2R account. Conversely, if deliberation only amplifies the effect of prior beliefs, this could be consistent with either politically motivated reasoning or accuracy motivated reasoning and thus would not provide unique support for the MS2R account.

Indeed, our results indicate that there was no significant three-way interaction between partisanship, argument type, and condition (study 1: *b* = 1.09, *P* = 0.770; study 2: *b* = −1.27, *P* = 0.790)—this is inconsistent with the MS2R account. In contrast, however—and qualifying the two-way interaction reported above in the model without prior beliefs—we find a significant (and very strong) three-way interaction between prior beliefs, argument type, and condition (study 1: *b* = −16.23, *P* < 0.0001; study 2: *b* = −14.12, *P* = 0.0007). Thus, prior beliefs (and not political partisanship) interact with reasoning during evidence evaluation.

To further understand the key three-way interaction between prior belief, condition, and argument type, we compared intuitive and deliberative responses separately for believers versus deniers (above versus below 0 on prior beliefs) for each argument type. We found that on the contrary arguments, “believers” were more likely to show lower agreement in the deliberative condition (study 1: *b* = −8.1, *P* < 0.0001; study 2: *b* = −8.52, *P* < 0.0001), while “deniers” were more likely to indicate higher agreement after deliberation (study 1: *b* = 13.23, *P* = 0.008; study 2: *b* = 10.1, *P* = 0.030). On the pro arguments, neither believers (study 1: *b* = 1.52, *P* = 0.275; study 2: *b* = 0.07, *P* = 0.960) nor deniers (study 1: *b* = 3.14, *P* = 0.530; study 2: *b* = 0.32, *P* = 0.950) significantly changed their agreement after deliberation. Further subsetting on Republicans, we found that Republican deniers showed significantly higher agreement with contra arguments (study 1: *b* = 14.34, *P* = 0.014; study 2: *b* = 13.84, *P* = 0.009), but Republican believers showed significantly lower agreement with contra arguments in the deliberative condition (study 1: *b* = −6.18, *P* = 0.009; study 2: *b* = −5.9, *P* = 0.030). This clearly demonstrates that deliberation did not amplify agreement for Republicans per se nor did it increase agreement with the scientific consensus per se, but rather that deliberation increased the coherence between the presented evidence and prior beliefs.

Finally, to avoid any potential selection bias arising from noncompliance with the time pressure manipulation (49), we replicated our main analyses while replacing any intuitive responses where the participant did not respond within the time limit (such that no response was recorded and thus was not included in the main analyses above) with their corresponding final response—that is, assuming that missed responses correspond to no effect of deliberation. The results were qualitatively equivalent (significant two-way interaction between the argument type and condition, study 1: *b* = −5.59, *P* = 0.001; study 2: *b* = −4.99, *P* = 0.007; after adding partisanship and beliefs in the models, we found no significant three-way interaction between partisanship, argument type, and condition, study 1: *b* = 0.64, *P* = 0.860; study 2: *b* = −0.9, *P* = 0.840; and a significant three-way interaction between prior belief, argument type, and condition, study 1: *b* = −15.72, *P* < 0.0001; study 2: *b* = −14.49, *P* < 0.001^3^).

We, therefore, found no positive evidence in unique support of the MS2R account. We did find support for the belief coherence account's predictions regarding the contra climate arguments, but—inconsistent with any of the accounts—we found no significant effects at all of deliberation on pro climate arguments. One potential explanation of this difference between argument types is that most participants may have already been exposed to the pro climate change arguments and, therefore, have stronger, and more quickly available, prior beliefs about those arguments, such that they would not need additional time to deliberate when evaluating them. Conversely, the contra climate arguments were likely to have been more novel, such that evaluating them required more deliberation. In other words, prior exposure to pro arguments might have (i) prevented deliberation to have an impact because people had their intuitive judgements aligned with their prior beliefs (so they had no reason to deliberate (19, 50)) or (ii) caused a floor/ceiling effect in the intuitive condition which prevented any detectable deliberation effect to arise. This is well illustrated by the fact that agreement scores are higher for believers than for deniers in the intuitive response condition for contra climate arguments (i.e. there is a misalignment between intuitive judgements and prior beliefs), while agreement scores are generally much lower for deniers for pro climate arguments (i.e. there is no misalignment between intuitive judgments and prior beliefs; see Figs. S4–S9 for raw averages and response distributions).

**Fig. 4.**
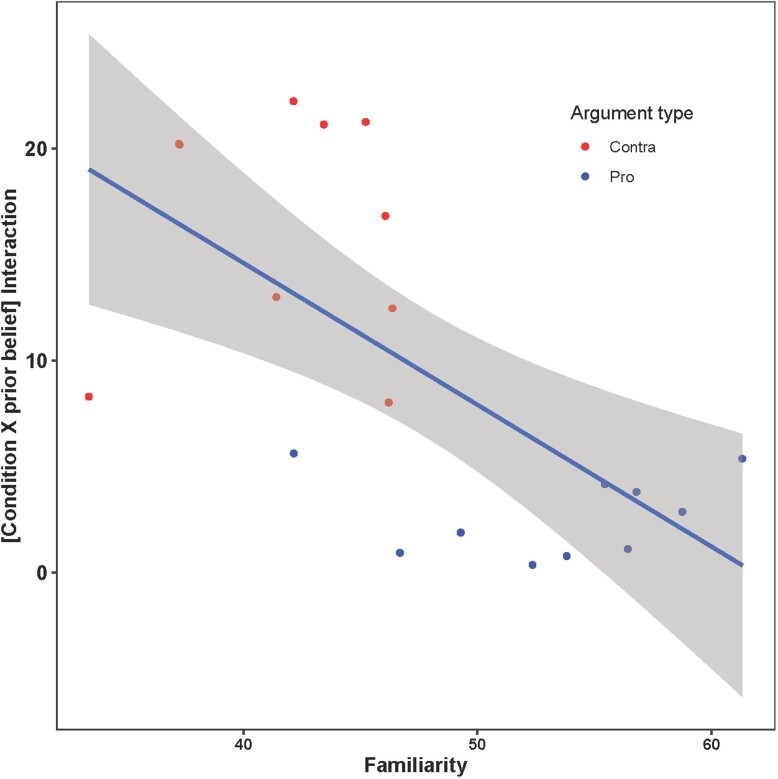
Deliberation magnifies the effect of prior beliefs to a greater extent for less familiar arguments. Shown is one dot per argument, with average out-of-sample familiarity rating on the *x*-axis and the coefficient on the interaction between condition and prior belief (indicating the extent to which deliberation magnifies the effect of prior beliefs) on the *y*-axis. Contra climate change arguments are shown in red; pro climate change arguments are shown in blue.

To further evaluate whether familiarity had an effect on deliberation's impact on argument evaluation, we conducted an additional experiment (Lucid sample, *N* = 4,733) in which we measured people's familiarity with each argument (on a scale from 0: absolutely not familiar, to 100: very familiar). Consistent with the above familiarity account, people were more familiar with pro climate (mean familiarity = 53.4) than contra climate items (mean familiarity = 41.9), *t* (4720.4) = −12.5, *P* < 0.0001. Even more importantly, we found a strong argument-level correlation between an argument's average familiarity and the coefficient on the interaction between deliberation and prior belief for that specific item (*r* = −0.64, *P* = 0.002; Fig. 4). This shows that deliberation magnified the effect of prior beliefs to a greater extent for more unfamiliar arguments—and thus that differences in familiarity may explain the asymmetry that we observed in our main studies between pro climate and contra climate arguments.

In sum, our results suggest that deliberation moves agreement with anticlimate change arguments toward participants’ preexisting prior beliefs about climate change, supporting the belief-coherence account of reasoning. Graphs on the means and distribution of responses for each response condition, party affiliation, prior belief, and argument type can be found in Supplementary material Section D and Figs. S4–S9.

## Discussion

Here, we investigated the role of reasoning in climate change (dis)belief, addressing two major limitations of prior work: We accounted for prior beliefs (which we demonstrate are correlated, but dissociable, from partisanship per se), and we experimentally manipulated the extent of reasoning rather than relying on correlations with individual differences in reasoning. Our results showed that once prior beliefs were accounted for, the apparent causal effect wherein deliberation increases reliance on partisan identities disappeared. Instead, deliberation led to improved coherence between the evaluation of novel climate arguments and one's preexisting beliefs about climate change, regardless of one's partisanship.

These results show that patterns typically taken as evidence for the MS2R account—i.e. that individual deliberative abilities are associated with increased political polarization about climate change (11)—do not actually provide any positive evidence in favor of the politically motivated reasoning account over alternative accounts. Such patterns could also be explained by accuracy-motivated reasoners with (even incidental) variation in their prior beliefs. More specifically, it may be the case that more deliberative people simply rely more on their prior beliefs when evaluating evidence (a strategy that is often rational (37)) rather than deferring to their identities per se. Our results indicate that people relied on their prior beliefs when deliberating about anticlimate messages and that this occurred regardless of their partisan identities. Thus, our results are consistent with the accuracy-motivated reasoning account and not a direct effect of identity as per the MS2R account. Nonetheless, it may be the case that partisan identities have an *indirect* effect on reasoning because prior beliefs are themselves influenced by identities (e.g. partisanship may have a direct effect on what sort of news media that people consume (51), which would influence prior beliefs and therefore subsequent evidence evaluation).

Although we specifically focused on politically motivated reasoning, more general (nonpolitical) motivations to defend one's preexisting beliefs could also potentially explain our results. That is, deliberation may help people to find rationalizations for why their existing beliefs are correct (so that, for example, they avoid the discomfort of having been wrong)—which would make them directionally motivated reasoners rather than accuracy-motivated reasoners (32). While our results are consistent with such a process, they also do not provide positive evidence in favor of it as it is not possible to distinguish between this rationalization and accuracy-motivated reasoning in this context. In short, a genuine attempt to accurately evaluate the evidence may appear like rationalization (and vice versa) absent additional information about an individual’s motivations. Indeed, it has even been argued that rationalization can itself be rational (52).

While our empirical results do not directly differentiate between these different accounts, it is our opinion that the accuracy-motivated account offers the best explanation for them. In the absence of positive evidence for MS2R (be it political or belief driven), we argue that it is more parsimonious to conclude that reasoning about climate change is guided by coherence with prior beliefs. Much prior work shows that coherence with priors guides reasoning in the same way as what we observe here but in situations completely unrelated to partisan identities or belief defense (such as in syllogistic/deductive reasoning) (36, 53–56). Thus, it seems more parsimonious to explain the current results using the accuracy motives that are widely agreed to operate in noncontroversial contexts, rather than invoking an additional directional motivation unique to controversial contexts.

Future work should approach this issue in the same experimental fashion that we approached deliberation in the current work, namely, by experimentally disentangling the effects of prior beliefs and partisan motivations. Furthermore, with respect to belief defense, we find that deliberation magnifies the effect of prior beliefs to a greater extent for less familiar arguments. If one assumes that specific preexisting beliefs are stronger for more familiar arguments, then, one should expect the opposite pattern to emerge under the belief defense account—i.e. climate change acceptance/denial would have a *stronger* interaction with deliberation when evaluating more *familiar* arguments. Instead, we find that deliberation increases belief-coherent reasoning most when people are evaluating *novel* arguments. This provides indirect evidence against the defense-of-existing-belief interpretation.

It is important to note that we do find evidence for direct effects of both partisanship and prior beliefs on argument evaluation, meaning that there is evidence that partisanship plays a role in climate change acceptance or denial that cannot be entirely explained by specific prior beliefs. However, in line with previous correlational findings (31, 37), we show causal evidence that deliberation only amplifies the direct effect of prior beliefs and not the direct effect of partisanship. Hence, one important aspect for future research would be to better understand how partisanship directly influences argument evaluation if not via deliberation.

The foregoing suggests that partisanship may influence our prior beliefs even without the need for deliberation, which could potentially support a more complex model of political motivations where they primarily influence intuitive priors, which then interact with a (potentially, but not always) corrective effect of deliberation. In any case, our experiment also does not allow us to identify what caused the differential priors that climate believers versus deniers brought into our study. It seems likely that exposure to differential information streams (e.g. right- versus left-leaning news channels) is a major contributor (57). It is also possible that political motivations influenced belief formation or updating at some earlier stage (that is, that prior beliefs mediate the effect of partisanship). Note that besides differences in knowledge, there are other factors that could influence individual differences in prior beliefs, such as elite cues, extreme weather events, or exposure to advocacy (58). It is also possible that potential cognitive asymmetries (e.g. differences in epistemic motivations) behind ideologies also play a role in prior belief formation (59). Shedding light on how partisan (or ideological) differences in priors emerge is an important direction for future work. In any case, regardless of how prior beliefs may have arisen, our results show that engaging in reasoning does not, in and of itself, lead to an increased direct effect of partisan alignment when people evaluate arguments relating to climate change.

While it is possible that the behavior that we observe is rational, of course, being “rational” (Bayesian) does not mean that deliberation will necessarily lead to greater belief in climate change. Indeed, deliberation only leads to lower belief in (incorrect) contra climate change arguments among people who already believe that climate change is real. In contrast, deliberation actually drives deniers further away from accuracy. Our results, therefore, suggest that simply getting people to think more carefully will not be sufficient to make people more likely to believe in the threat of climate change.

At the same time, our results also suggest that things may not be as bleak as suggested by the MS2R account. Our findings are consistent with the possibility that people are engaging in a good-faith effort to assess new information accurately and relying on their prior beliefs to guide such judgments. If this is indeed what is happening, then, educational interventions could—in the long run—move people's priors and increase agreement with the scientific consensus. Consistent with this interpretation, there is substantial evidence that informing people about climate change to increase knowledge about issues surrounding climate change—that is, to shift people's prior beliefs (25, 25–27, 60, 61)—is an effective technique for mitigating climate disbelief. Although our results imply that influencing priors over a long time should have lasting changes on how people evaluate novel arguments about climate change, there is a considerable disagreement about what type of information is most helpful (27, 62–65) and how long the changes in priors can last (66)—these will be important directions for future research. Our results suggest that once this knowledge is obtained, inducing deliberation may help people resist climate misinformation and reduce their likelihood of engaging in science denial.

Our research also speaks to the role of cognitive sophistication in the success of deliberation—an aspect that has been key to recent developments in the dual-process literature (29, 42, 43, 67). Two distinct factors that are often mentioned as key to the success of deliberation are fluid intelligence (i.e. a mix of flexible thinking skills and the ability to solve novel, often abstract problems) and crystallized intelligence (i.e. acquired knowledge and skills) (67, 68). This is very similar to the dual importance of deliberation and prior beliefs in our findings. Specifically, the finding that deliberation can increase accuracy points to the importance of fluid intelligence whereas the finding that specific prior beliefs have an influence on the role of deliberation indicates that crystallized intelligence (i.e. the quality of prior beliefs) is also critical (69). Improving decision-making requires a multipronged approach that targets different elements of what it means to be a sophisticated reasoner.

There are some limitations of our work that it is important to acknowledge. First, climate change belief was assessed with only two questions. Although these measured prior stances on two important dimensions of climate change (that it is caused by human activity and that it poses a significant threat to humanity), more extensive measures of climate disbelief might have led to stronger results. Future experiments should keep that in mind. Furthermore, we only examined prior beliefs about climate change. Future work should also examine other relevant beliefs, such as beliefs about the reliability of science and the trustworthiness of scientists, and whether they influence deliberation and belief formation. Second, although Lucid uses quota matching on age, gender, ethnicity, and geographic region, our sample should not be considered fully representative of the US population. It should be noted, however, that our sample *is* ideologically balanced—there was an almost identical number of Republicans and Democrats—and our estimate of prior belief in anthropogenic climate change is reasonably close to the national average: According to US-wide, nationally representative surveys (5), 20% of Americans believe that human activity contributes no or very little to global warming (i.e. the people who would constitute as “deniers” in our sample), while we found 15.1%. Relatedly, we only recruited participants from the United States, while climate change is obviously a global issue. Thus, it is important for future work to examine how our results generalize to other countries with differing political cultures.

Another limitation involves the abstracted context (a survey experiment) in which we presented the pro and contra climate arguments. It would be fruitful for future work to examine how richer social contexts influence reasoning. Indeed, for example, there is evidence that when evaluating messages about climate change, people are also affected by the messenger such that information about the impacts of climate change had a bigger impact on the beliefs of Republican voters if it came from Republican leaders (70). People, therefore, should receive information from sources that they find credible; otherwise, the message might fail to shift deniers’ priors thereby making long-term belief change unlikely. A potential solution is to recruit individuals who are trusted by climate deniers or to improve the credibility of mediums willing to communicate accurate information.

The data presented here do not provide support for the dominant MS2R account of climate misbelief. Instead, our results are as consistent with climate deniers making a good-faith effort to form accurate beliefs, but operating with corrupted prior beliefs, as they are with MS2R. If this accuracy-motivated explanation is correct, the blame for climate denial would then rest far more on the producers and distributors of climate misinformation than on the public. This emphasizes the key role of effective climate communication—disseminating accurate information about climate change in an engaging manner is essential for winning the battle against climate denial.

## Materials and methods

### Participants

#### Study 1

In total, 1,007 participants were recruited from Lucid. In the two-response paradigm (intuitive condition), 629 participants (315 females, 308 males, and 6 others; mean age = 45.34 years; SD = 16.9 years) took part, while in the one-response study (deliberative condition), 378 people (197 females, 180 males, and 1 others; mean age = 45.2 years; SD = 16.6 years) participated. In the two-response condition, 314 people were Democrats, and 314 were Republicans, and in the one-response test, 194 were Democrats and 179 were Republicans.

#### Study 2

In total, 1,266 participants were recruited from Lucid who passed our first screening question (from a total of 1,489 participants who clicked on the experiment link, 12 did not consent, and an additional 211 did not pass the first screening question and were not allowed to participate; these participants produced no data whatsoever). In the two-response paradigm (intuitive condition), 758 participants (387 females, 341 males, and 8 others; mean age = 45 years; SD = 17.4 years) took part, while in the one-response study (deliberative condition), 508 people (251 females, 249 males, and 1 others; mean age = 45.7 years; SD = 17.2 years) participated. In the two-response condition, 407 people were Democrats, and 318 were Republicans, and in the one-response test, 306 were Democrats and 192 were Republicans.

This research was approved by The MIT Committee on the Use of Human Experimental Subjects. Before the experiment, we obtained informed consent from all participants.

Preregistration for study 2 can be found at https://osf.io/4axr6/.

### Materials and procedures

#### Climate arguments

Altogether, participants were presented with six arguments in a randomized order. All the arguments that we used in this study were taken from *procon.org* and can be seen in the Supplementary Material Section A (Table S1). Half of these arguments were supporting arguments for anthropogenic climate change. These were scientific arguments, mostly explaining how climate change affects our environment or how human activity causes climate change, all related to climate change, while the other half of the arguments argued against that humans causing climate change or that it would have bad consequences. All of these arguments were presented in a nonpartisan manner; there were no party cues or politicians mentioned whatsoever. Arguments were content-counterbalanced, and participants never saw the pro and contra versions of a given content. Participants were presented with only one of the two versions of one argument but were always presented with three pro and three contra arguments. After each argument, we asked people “How much do you agree with this argument (0 = completely disagree, 100 = completely agree)?” Participants could give a response on a slider. To make sure that the position of the handler (the button participants could pull and push with their mouse to indicate their response) on the slide does not bias participants, the handler was not shown until participants first clicked on the slide.

#### Dot matrix task (cognitive load)

As we wanted to minimize the impact of system 2 deliberation in the initial response stage of the two-response experiment, participants were presented with a cognitive load. The rationale here is simple: Insofar as system 2 deliberation depends upon working memory to operate (17), restricting working memory capacity should increase reliance on system 1 intuitions (which do not depend upon working memory). As in other two-response paradigm experiments (e.g. (71)), we used a dot matrix task (72) which has been shown to decrease analytical engagement in many tasks including probabilistic reasoning (73) and moral reasoning (74). In this task, before the argument is presented, participants are presented with a 4 × 4 matrix, with five dots in it, and they are instructed to memorize the dot pattern. After the initial response stage, participants are presented with a set of four matrices and they were asked to select the matrix that was presented to them in the beginning. After they made a decision, they were given feedback as to whether they selected the correct one or not. In cases where the participant failed to select the correct option, they were asked to pay more attention on the subsequent trials. Load was not applied during the final response stage (i.e. they did not have to learn and remember a dot matrix while giving a response in the final response stage).

#### Response deadline and reading pretest

To further assure the intuitive nature of the initial response, participants had to indicate their answer under a strict response deadline (47, 71, 75). The rationale here is that system 2 is argued to be relatively slower to produce a response than system 1 processing. Hence, by presenting participants with a deadline, we decrease the probability of system 2 engagement. To help us define the deadline, we run a reading pretest with *N* = 101 participants (35 females and 66 males; mean age = 35.24 years; SD = 12 years) on MTurk. Participants were presented with the same arguments as in the actual experiment. They were instructed to simply read the material and then click on the “Next” button. We then logarithmically transformed RT data and back transformed the data, thereby calculating the geometric mean. We found that, on average, people took 27.3 s (SD = 34.02) to read the headlines (after excluding all trials three times the SD above or below the mean). Hence, we decided to set the response deadline to the closest integer of the average reading time: 28 s.

#### Partisanship and prior beliefs

We measured participants’ political partisanship with a single question: “Which of the following best describes your political preference (1—strongly Democratic, 6—strongly Republican)?” To measure specific prior beliefs about climate change, in study 1, we asked the following question: “How much do you agree with the idea that human activity causes global changes in climate (completely agree, somewhat agree, somewhat disagree, and completely disagree)?” In study 2, additionally, we also asked participants: “How significant is the risk climate change poses on humanity^4^ (very significant, somewhat significant, somewhat insignificant, and very insignificant)?” In study 2, we calculated the average of these two measures for every participant to create a “prior belief” score which we will later apply in the analysis. These questions reflect the position that our climate arguments argue for or against the best; hence, they constitute as the best way to measure specific prior belief in this specific context. We added the “risk” question in study 2 precisely to make our belief scale more precise and better reflect the positions of the arguments. In study 1, these questions were asked after climate change arguments, at the end of the experiment, while in study 2, right at the beginning of the experiment, right before the instructions.

#### Two-response paradigm procedure (referred to as “intuitive condition”)

Before the instructions, participants in study 2 were presented two screening questions to screen careless respondents (study 1 did not include screeners). Participants in the treatment were then told that they are going to be presented with arguments twice. The literal instructions were as follows:

“Welcome to the experiment!

Please read these instructions carefully!

This experiment is composed of 6 questions and a couple of practice questions. It will take about 15 minutes to complete and it demands your full attention. You can only do this experiment once.

In this experiment, you will be presented with different arguments regarding climate change and its potential effects. You will be asked you to indicate how much you agree with the argument on a scale from 0 = completely disagree to 100 = completely agree. We want to know what your initial, intuitive decision is and how you respond after you have thought about the problem for some more time. Hence, as soon as the problem is presented, we will ask you to enter your initial response. We want you to respond with the very first answer that comes to mind. You don’t need to think about it. Just give the first answer that intuitively comes to mind as quickly as possible. Next, the argument will be presented again and you can take all the time you want to actively reflect on it. Once you have made up your mind you can enter your final response. You will have as much time as you need to indicate your second response.

To assure that the initial response is really intuitive, you will have 28 seconds to give a response. 3 second before the deadline passes, the background will turn yellow to warn you.

In sum, keep in mind that it is really crucial that you give your first, initial response as fast as possible. Afterwards, you can take as much time as you want to reflect on the problem and select your final response.

Please confirm below that you read these instructions carefully and then press the “Next” button. We will start with a couple of practice problems.”

After the instructions, participants were presented with a practice problems. After this, they were presented with dot matrix practice problems in which they were only asked to memorize the dot pattern and try to select the correct one out of the four matrices presented afterwards. Then, they were presented with a new practice problem, in which they had to give an initial response under load (i.e. keeping the dot matrix in mind while reading and evaluating the argument).

Each trial started with a presentation of a fixation cross (a cross in the middle of the screen where the dot matrix would appear—to direct people's attention to the place in which the matrix would appear) which stayed on screen for 1,000 ms. After it disappeared, the dot matrix was presented for 2,000 ms. Then, the argument was presented and participants had 28 s to give a response; 3 s before the deadline, the background turned yellow, to warn participants of the approaching deadline. In case that they did not manage to give a response before the deadline passed, they received a message saying “You did not enter your response before the deadline. Try to respond within the deadline on the next trials. No big deal if you're not totally sure. Just enter your very first intuitive answer. You get more time to reflect on your answer afterwards.” After the initial response or the message, they were presented with the dot matrix question and had to select the pattern that they were presented with. They received feedback on whether or not they selected the correct pattern. In case they did not, they were warned to try to focus on recalling the correct pattern in subsequent rounds. After the feedback, they were presented with the same argument again and were asked to give a final response.

Next, participants were asked to complete a six-item version of the CRT: first, they were presented with content-modified versions (30) of the original three CRT items (44) after which they were presented with a newer three-item nonnumerical CRT (76).

In the two-response paradigm, there is the potential for the second response to be anchored on the first response (47). That is, the effect of deliberation may be underestimated due to having explicitly stated one's intuitive response initially (and then being anchored on that response). Therefore, we focus our main text discussion on comparing the first (intuitive) response with the one-response control condition (described below). We report results for the second response in Supplementary material Section C and Table S3, where, indeed, we do find evidence of anchoring (comparing second response to control) but nonetheless generally replicate our main results when testing the effect of deliberation by comparing the initial response to the final response^5^.

#### One-response paradigm procedure (referred to as “deliberative condition”)

In this (control) condition, participants were presented with each argument only once. These are the literal instructions that participants received at the beginning of this experiment:

“Welcome to this experiment!

In this experiment, you will be presented with different arguments regarding climate change and its potential effects. You will be asked you to indicate how much you agree with the argument on a scale from 0 = completely disagree to 100 = completely agree. Then, you will be presented with the same problem again and we will ask you three other questions. Please carefully read through the arguments before responding.

You will be presented with six arguments. The experiment will take about 15 minutes to complete, and will demand your full attention.

Press ‘Next’ to continue!”

In each trial, after a brief fixation cross-period (1 s), the argument appeared and they were asked to give a response without any (load or deadline) constraints. In short, participants in this condition were simply given the “final response” portion of the two-response paradigm. At the end of this experiment, participants were presented with the same CRT, science knowledge, and demographic questionnaires as in the two-response version.

#### Exclusion

For study 1, we only excluded trials in which the initial trial was missed, and hence, 6.1% of trials were excluded. We first excluded participants who did not pass our second screening question (note that people who did not pass the first screening question, 211 participants altogether, were not allowed to participate in the experiment); from the two-response experiment, we excluded 164 people, and from the one-response version, we excluded 125 participants. As stated in the preregistration, we excluded all trials in which participants did not manage to give an initial response in time (hence, there was no data recorded). The only exception is when we compared baseline and final responses in which analysis we included all the data. Some participants also did not finish the experiment and quit after giving an initial response. These data were also excluded from the initial versus final analysis but not from the rest. Altogether, 11.6% of trials were excluded from analysis for missing data.

#### Statistical analysis

The maximal model converged in none of the cases. In all models, we included argument condition (0.5—contra, −0.5—pro) and condition (initial, final, and baseline). As stated in the preregistration, belief was coded as a numeric variable from −1 (strong disbelief) to 1 (strong belief) and partisanship similarly as a numeric, from −1 (strongly Democratic) to 1 (strongly Republican). We compared conditions with each other, always including only two of them in the models (e.g. initial versus final). Table 2 shows all the fixed and random effects that were included in the separate models. In all models, the intercept was allowed to vary over subjects and item contents. We published unstandardized beta coefficients; for the standardized betas, see Table S4.

## Supplementary Material

pgad100_Supplementary_Data

## Data Availability

Data is made open and available at https://osf.io/6fcre/.

## Footnotes

1 Rationality here and throughout this manuscript refers to the normative nature of the thought process. The outcome of such a process can nevertheless be inaccurate. Imagine a 4-year-old child who is repeatedly told by his parents and grandparents that Santa Claus is real. If another kid, who is plainly less trustworthy than known adults, states that Santa is not real, it would not be irrational (absent additional exculpatory information) to maintain belief in Santa Claus. Hence, given strong existing prior beliefs, rational processes can arrive at the wrong conclusion.

2 To define the response deadline, we conducted a reading pretest experiment, in which we simply asked participants to read the material. The response deadline is the average of the reading times in this pretest.

3 For completeness, we also added an analysis to the supplementary testing whether demographics and other variables affect exclusion (Table S5). Exclusion slightly correlates with age (more excluded from older), gender (more excluded from males), and politics (slightly more excluded from Democrats), but cognitive ability, argument type, prior belief, argument type*politics, and argument type*prior belief interaction effects were not significant.

4 In the actual experiment, there was a grammatical error in this question: “How significant is the risk climate change pose on humanity?”

5 In study 1, we fully replicate the findings of the between-subject experiment. In study 2, we find that both deliberation condition and argument type significantly interact with the deliberation condition. This is probably caused by decreased prior belief effect sizes due to anchoring. We also conducted a pooled analysis that replicates the findings of the main experiments fully.
