# Supplementary material for: Reasoning about climate change
Source: PNAS Nexus. 2023 May 2;2(5):pgad100. doi: 10.1093/pnasnexus/pgad100 (PMC10153421; doi:10.1093/pnasnexus/pgad100)
Supplement: pgad100_Supplementary_Data [file pgad100_supplementary_data.docx]

**Supplementary Materials for “Reasoning about Climate Change”**

Bence Bago, David G. Rand, Gordon Pennycook

1. **Climate change arguments**

**Table S1.** Table shows all the arguments that were used in the main experiment

| Contrary to climate change | Pro climate change |
| --- | --- |
| Earth’s climate has always warmed and cooled, and the 20th century rise in global temperature is within the bounds of natural temperature fluctuations over the past 3,000 years. Although the planet has warmed 1-1.4°F over the 20th century, it is within the +/- 5°F range of the past 3,000 years. A 2003 study by researchers at the Harvard-Smithsonian Center for Astrophysics found that "many records reveal that the 20th century is probably not the warmest nor a uniquely extreme climatic period of the last millennium."    A 2005 study published in Nature found that "high temperatures - similar to those observed in the twentieth century before 1990 - occurred around AD 1000 to 1100" in the Northern Hemisphere. A 2013 study published in Boreas found that summer temperatures during the Roman Empire and Medieval periods were "consistently higher" than temperatures during the 20th century. According to a 2010 study in the Chinese Science Bulletin, the recent global warming period of the 20th century is the result of a natural 21-year temperature oscillation, and will give way to a "new cool period in the 2030s." | Average temperatures on earth have increased at a rate far faster than can be explained by natural climate changes. A 2008 study compared data from tree rings, ice cores, and corals over the past millennium with recent temperature records. The study created the famous "hockey stick" graph, showing that the rise in earth's temperature over the preceding decade had occurred at a rate faster than any warming period over the last 1,700 years. In 2012 the Berkeley scientists found that the average temperature of the earth’s land increased 2.5°F over 250 years (1750-2000), with 1.5°F of that increase in the last 50 years.    Lead researcher Richard A. Muller, PhD, said "it appears likely that essentially all of this increase [in temperature] results from the human emission of greenhouse gases." In 2013, a surface temperature study published in Science found that global warming over the past 100 years has proceeded at a rate faster than at any time in the past 11,300 years. According to the IPCC’s 2014 Synthesis Report, human actions are "extremely likely" (95-100% confidence) to have been the main cause of 20th century global warming, and the surface temperature warming since the 1950s is "unprecedented over decades to millennia." |
| Even if humans are creating a slightly warmer climate, it’s not necessarily a bad thing. The underlying assumption that virtually all climate alarmists operate under is that the warming Earth is experiencing now is harmful, destructive and dangerous, but there is much evidence to suggest that moderate warming benefits most plants, animals and humans. We know, for instance, that plants grow significantly better with higher carbon-dioxide concentrations, which is why many greenhouses pump additional CO2 into their buildings.    It’s also been confirmed by multiple studies that greening has increased in recent decades — and likely because of higher carbon-dioxide concentrations. According to a study by Martin Brandt et al., published in the journal Nature Ecology & Evolution in 2017, 36 percent of the continent of Africa became greener over the 20-year period from 1992 to 2011, while only 11 percent became “less green.” Interestingly, the researchers found the increased greening was likely “driven” by higher carbon-dioxide levels and precipitation, and the decreased greening was largely a result of humans cutting down vegetation. | There are some clear disadvantages to a warmer climate:  • Agriculture depends on a supply of steady water, and floods and droughts will disrupt that.  • The polar regions may thaw, but the soil up there is poor and that portion of the planet receives little direct sunlight.  • Fewer people will freeze to death, but more people will die from the heat.  • Disease-carrying insects like mosquitoes will thrive in warmer temperatures and carry diseases to new places, an effect we are already seeing. |
| Global warming and cooling are primarily caused by fluctuations in the sun’s heat (solar forcing), not by human activity. Over the past 10,000 years, solar minima (reduced sun spot activity) have been "accompanied by sharp climate changes." Between 1900 and 2000 solar irradiance increased 0.19%, and correlated with the rise in US surface temperatures over the 20th century. According to a 2007 study published in Energy & Environment, "variations in solar activity and not the burning of fossil fuels are the direct cause of the observed multiyear variations in climatic responses."    In a 2012 study by Willie Soon, PhD, Physicist at the Harvard-Smithsonian Center for Astrophysics, a strong correlation between solar radiation and temperatures in the Arctic over the past 130 years was identified. According to a 2012 study published in the Journal of Atmospheric and Solar-Terrestrial Physics, "up to 70% of the observed post-1850 climate change and warming could be associated to multiple solar cycles." | Natural changes in the sun’s activity cannot explain 20th century global warming. According to a Dec. 2013 study in Nature Geoscience, the sun has had only a "minor effect" on the Northern Hemisphere climate over the past 1,000 years, and global warming from human-produced greenhouse gases has been the primary cause of climate change since 1900. Another 2013 study found that solar activity could not have contributed to more than 10% of the observed global warming over the 20th century.    Measurements in the upper atmosphere from 1979-2009 show the sun's energy has gone up and down in cycles, with no net increase. According to a 2013 IPCC report, there is "high confidence" (8 out of 10 chance) that changes in the sun's radiation could not have caused the increase in the earth's surface temperature from 1986-2008. Although warming is occurring in the lower atmosphere (troposphere), the upper atmosphere (stratosphere) is actually cooling. If the sun were driving global warming, there would be warming in the stratosphere also, not cooling. |
| Sea levels have been steadily rising for thousands of years, and the increase has nothing to do with humans. A 2014 report by the Global Warming Policy Foundation found that a slow global sea level rise has been ongoing for the last 10,000 years. When the earth began coming out of the Pleistocene Ice Age 18,000 years ago, sea levels were about 400 feet lower than they are today and have been steadily rising ever since.    According to Professor of Earth and Atmospheric Sciences at the Georgia Institute of Technology, Judith Curry, PhD "it is clear that natural variability has dominated sea level rise during the 20th century, with changes in ocean heat content and changes in precipitation patterns." Freeman Dyson, Emeritus Professor of Mathematical Physics and Astrophysics at the Institute for Advanced Study at Princeton University, has stated that there is "no evidence" that rising sea levels are due to anthropogenic climate change. | Sea levels are rising at an unprecedented rate due to global warming. As human-produced greenhouse gases warm the planet, sea levels are rising due to thermal expansion of warming ocean waters as well as melt water from receding glaciers and the polar ice cap. According to the IPCC, there has been a "substantial" human contribution to the global mean sea-level rise since the 1970s, and there is "high confidence" (8 out of 10 chance) that the rate of sea-level rise over the last half century has accelerated faster than it has over the previous 2,000 years.    A 2006 study found that "significant acceleration" of sea-level rise occurred from 1870 to 2004. Between 1961 and 2003 global sea levels rose 8 inches. An Oct. 2014 study published in the Proceedings of the National Academy of Sciences concluded that the rate of sea level rise over the past century is unprecedented over the last 6,000 years. A separate Oct. 2014 study said that the global sea level is likely to rise 31 inches by 2100, with a worst case scenario rise of 6 feet. Climate Central predicts that 147 to 216 million people live in areas that will be below sea level or regular flood areas by the end of the century if human-produced greenhouse gas emissions continue at their current rate. |
| Glaciers have been growing and receding for thousands of years due to natural causes, not human activity. The IPCC predicted that Himalayan glaciers would likely melt away by 2035, a prediction they disavowed in 2010. In 2014 a study of study of 2,181 Himalayan glaciers from 2000-2011 showed that 86.6% of the glaciers were not receding. According to a 2013 study of ice cores published in Nature Geoscience, the current melting of glaciers in Western Antarctica is due to "atmospheric circulation changes" that have "caused rapid warming over the West Antarctic Ice Sheet" and cannot be directly attributed to human caused climate change.    According to one of the study authors, "[i]f we could look back at this region of Antarctica in the 1940s and 1830s, we would find that the regional climate would look a lot like it does today, and I think we also would find the glaciers retreating much as they are today." According to Christian Schlüchter, Professor of Geology at the University of Bern, 4,000 year old tree remains have been found beneath retreating glaciers in the Swiss Alps, indicating that they were previously glacier-free. According to Schlüchter, the current retreat of glaciers in the Alps began in the mid-19th century, before large amounts of human caused CO2 had entered the atmosphere. | Glaciers are melting at unprecedented rates due to global warming, causing additional climate changes. About a quarter of the globe's glacial loss from 1851-2010, and approximately two thirds of glacial loss between 1991-2010, is attributable directly to global warming caused by human-produced greenhouse gases. According to the National Snow and Ice Data Center, global warming from human-produced greenhouse gases is a primary cause of the "unprecedented" retreat of glaciers around the world since the early 20th century. Since 1980 glaciers worldwide have lost nearly 40 feet (12 meters) in average thickness.    According to a 2013 IPCC report, "glaciers have continued to shrink almost worldwide" over the prior two decades, and there is "high confidence" (about an 8 out of 10 chance) that Northern Hemisphere spring snow continues to decrease. If the glaciers forming the Greenland ice sheet were to melt entirely, global sea levels could increase by up to 20 feet. Melting glaciers also change the climate of the surrounding region. With the loss of summer glacial melt water, the temperatures in rivers and lakes increase. According to the US Geological Service, this disruption can include the "extinction of temperature sensitive aquatic species." |
| More than one thousand scientists disagree that human activity is primarily responsible for global climate change. In 2010 Climate Depot released a report featuring more than 1,000 scientists, several of them former UN IPCC scientists, who disagreed that humans are primarily responsible for global climate change. The Cook review of 11,944 peer-reviewed studies found 66.4% of the studies had no stated position on anthropogenic global warming, and while 32.6% of the studies implied or stated that humans are contributing to climate change, only 65 papers (0.5%) explicitly stated "that humans are the primary cause of recent global warming."    A 2012 Purdue University survey found that 47% of climatologists challenge the idea that humans are primarily responsible for climate change and instead believe that climate change is caused by an equal combination of humans and the environment (37%), mostly by the environment (5%), or that there’s not enough information to say (5%). In 2014 a group of 15 scientists dismissed the US National Climate Assessment as a "masterpiece of marketing," that was "grossly flawed," and called the NCA’s assertion of human-caused climate change "NOT true." | Overwhelming scientific consensus says human activity is primarily responsible for global climate change. The 2010 Anderegg study found that 97-98% of climate researchers publishing most actively in their field agree that human activity is primarily responsible for global climate change. The study also found that the expertise of researchers unconvinced of human-caused climate change is "substantially below" that of researchers who agree that human activity is primarily responsible for climate change.    The 2013 Cook review of 11,944 peer-reviewed studies on climate change found that only 78 studies (0.7%) explicitly rejected the position that humans are responsible for global warming. A separate review of 13,950 peer-reviewed studies on climate change found only 24 that rejected human-caused global warming. A survey by German Scientists Bray and Von Storch found that 83.5% of climate scientists believe human activity is causing "most of recent" global climate change. A separate survey in 2011 also found that 84% of earth, space, atmospheric, oceanic, and hydrological scientists surveyed said that human-induced global warming is occurring. |
| Rising levels of atmospheric CO2 do not necessarily cause global warming, which contradicts the core thesis of human-caused climate change. Earth's climate record shows that warming has preceded, not followed, a rise in CO2. According to a 2003 study published in Science, measurements of ice core samples show that over the last four climactic cycles (past 240,000 years), periods of natural global warming preceded global increases in CO2.    In 2010 the Proceedings of the National Academy of Sciences published a study of the earth's climate 460-445 million years ago which found that an intense period of glaciation, not warming, occurred when CO2 levels were 5 times higher than they are today. According to ecologist and former Director of Greenpeace International Patrick Moore, PhD, "there is some correlation, but little evidence, to support a direct causal relationship between CO2 and global temperature through the millennia." | The rise in atmospheric CO2 over the last century was clearly caused by human activity, as it occurred at a rate much faster than natural climate changes could produce. Over the past 650,000 years, atmospheric CO2 levels did not rise above 300 ppm until the mid-20th century. Atmospheric levels of CO2 have risen from about 317 ppm in 1958 to 415 ppm in 2019. CO2 levels are estimated to reach 450 ppm by the year 2040.    According to the Scripps Institution of Oceanology, the "extreme speed at which carbon dioxide concentrations are increasing is unprecedented. An increase of 10 parts per million might have needed 1,000 years or more to come to pass during ancient climate change events." Some climate models predict that by the end of the 21st century an additional 5°F-10°F of warming will occur. |
| Increased hurricane activity and other extreme weather events are a result of natural weather patterns, not human-caused climate change. According to a 2013 report from the Tropical Meteorology Project at Colorado State University, the increase in human-produced CO2 over the past century has had "little or no significant effect" on global tropical cyclone activity.    The report further states that specific hurricanes, including Sandy, Ivan, Katrina, Rita, Wilma, and Ike, were not a direct consequence of human-caused global warming. Between 1995-2015 increased hurricane activity (including Katrina) was recorded, however, according to the NOAA, it was not the result of human-induced climate change; it was the result of cyclical tropical cyclone patterns, driven primarily by natural ocean currents. Many types of recorded extreme weather events over the past half-century have actually become less frequent and less severe.    Professor of Earth and Atmospheric Sciences at the Georgia Institute of Technology, Judith Curry, PhD, states that she is "unconvinced by any of the arguments that I have seen that attributes a single extreme weather event, a cluster of extreme weather events, or statistics of extreme weather events" to human-caused climate change. Richard Lindzen, PhD, Emeritus Professor of Meteorology at the Massachusetts Institute of Technology, also states that there is a lack of evidence connecting extreme weather events such as hurricanes, tornadoes, droughts, or floods, to human-caused global warming. | Dramatic changes in precipitation, such as heavier storms and less snow, are another sign that humans are causing global climate change. As human-produced greenhouse gases heat the planet, increased humidity (water vapor in the atmosphere) results. Water vapor is itself a greenhouse gas. In a process known as a positive feedback loop, more warming causes more humidity which causes even more warming.    Higher humidity levels also cause changes in precipitation. According to a 2013 report published in the Proceedings of the National Academy of Sciences, the recorded changes in precipitation over land and oceans "are unlikely to arise purely due to natural climate variability." Higher temperatures from global warming are also causing some mountainous areas to receive rain rather than snow. According to researchers at the Scripps Institution of Oceanography, up to 60% of the changes in river flow, winter air temperature, and snow pack in the western United States (1950-1999) were human-induced.    Since 1991, heavy precipitation events have been 30% above the 1901-1960 average in the Northeast, Midwest, and upper Great Plains regions. A 2015 study found that global warming caused by human actions has increased extreme precipitation events by 18% across the globe, and that if temperatures continue to rise an increase of 40% can be expected. |
| The acidity levels of the oceans are within past natural levels, and the current rise in acidity is a natural fluctuation, not the result of human caused climate change. The pH of average ocean surface water is 8.1 and has only decreased 0.1 since the beginning of the industrial revolution (neutral is pH 7, acid is below pH 7). In 2010 Science published a study of ocean acidity levels over the past 15 million years, finding that the "samples record surface seawater pH values that are within the range observed in the oceans today."    Increased atmospheric CO2 absorbed by the oceans results in higher rates of photosynthesis and faster growth of ocean plants and phytoplankton, which increases pH levels keeping the water alkaline, not acidic. According to a 2010 paper by the Science and Public Policy Institute, "our harmless emissions of trifling quantities of carbon dioxide cannot possibly acidify the oceans." | Ocean acidity levels are increasing at an unprecedented rate that can only be explained by human activity. As excess human-produced CO2 in the atmosphere is absorbed by the oceans, the acidity level of the water increases. Acidity levels in the oceans are 25-30% higher than prior to human fossil fuel use. According to a 2014 US Government Accountability Office (GAO) report, oceans have absorbed about 30% of the CO2 emitted by humans over the past 200 years, and ocean acidity could rise approximately 100-200 percent above preindustrial levels by 2100.    According to a 2013 report from the World Meteorological Organization, the current acceleration in the rate of ocean acidification "appears unprecedented" over the last 300 million years. High ocean acidity levels threaten marine species, and slows the growth of coral reefs. According to a 2014 report by the Convention on Biological Diversity, "it is now nearly inevitable" that within 50-100 years continued human produced CO2 emissions will increase ocean acidity to levels that "will have widespread impacts, mostly deleterious, on marine organisms and ecosystems." |
| CO2 is already saturated in earth’s atmosphere, and more CO2, manmade or natural, will have little impact on climate. As CO2 levels in the atmosphere rise, the amount of additional warming caused by the increased concentration becomes less and less pronounced. According to Senate testimony by William Happer, PhD, Professor of Physics at Princeton University, "[a]dditional increments of CO2 will cause relatively less direct warming because we already have so much CO2 in the atmosphere that it has blocked most of the infrared radiation that it can.    The technical jargon for this is that the CO2 absorption band is nearly 'saturated' at current CO2 levels." According to the Heartland Institute's 2013 Nongovernmental International Panel on Climate Change (NIPCC) report, "it is likely rising atmospheric CO2 concentrations will have little impact on future climate." | The specific type of CO2 that is increasing in earth’s atmosphere can be directly connected to human activity. CO2 produced by burning fossil fuels such as oil and coal can be differentiated in the atmosphere from natural CO2 due to its specific isotopic ratio.    According to the Intergovernmental Panel on Climate Change (IPCC), 20th century measurements of CO2 isotope ratios in the atmosphere confirm that rising CO2 levels are the result of human activity, not natural processes such as ocean outgassing, volcanic activity, or release from other "carbon sinks." US greenhouse gas emissions from human activities in 2012 totaled 6.5 million metric tons, which is equivalent to about 78.3 billion shipping containers filled with greenhouse gases. |

1. **Correlational evidence**

**Methods**

**Correlational study 1a**

*Participants.* In total, 300 participants (112 females, 186 males and 1 others, Mean age = 35.2 years, SD = 10.6 years) took part in this study, who were recruited through MTurk, and completed the experiment online. In total, 183 people were Democrats, and 116 were Republicans.

**Materials & Procedure**

*Climate arguments.* Altogether, participants were shown 12 arguments in a randomised order (6 contra and 6 pro). Note that two of these arguments were not included in the two response experiments.

Procedure. At the beginning of the experiment, participants were presented with the following instructions:

***“Welcome to this experiment!***

*In this experiment, you will be presented with different arguments regarding climate change and its potential effects. You will be asked you to indicate how much you agree with the argument on a scale from 0 = completely disagree to 100 = completely agree. Then, you will be presented with the same problem again and we will ask you three other questions. Please carefully read through the arguments before responding.*

*You will be presented with 12 arguments. The experiment will take about 11 minutes to complete, and will demand your full attention.*

*Press 'Next' to continue!”*

After they finished responding to the arguments, participants were presented with a 6 item version of the cognitive reflection test, that is widely used to measure reflective abilities. Next, they were presented with a series of question to measure political knowledge and at the end, a series of standard demographic questions, regarding gender, age, political preferences. Most notably, to measure partisan preferences, we used the question: “Which of the following best describes your political preference? (Strongly Democrat/Democrat/Lean Democrat/ Lean Republican/Republican/Strong Republican)”, and to measure belief in anthropogenic climate change, we used the question: “How much do you agree with the idea that human activity causes global changes in climate? (Completely agree/Somewhat agree/Somewhat disagree/Completely disagree)”. Additionally, we also measured ideology with two questions; one asking about economic and another asking about social ideology: “On social/economic issues, I am: Strongly Liberal/Somewhat Liberal/Moderate/Somewhat Conservative/Strongly Conservative).

*Statistical analysis*. We used mixed effect regression models. Political preference was coded by a numeric variable (-3, -2, -1, 1, 2, 3; from Democrat to Republican), and climate belief was coded by another variable (-2, -1, 1, 2; from believer to non-believer)., and argument type was also coded by a dummy (-0.5 - pro, 0.5 - contra). For ideology, we averaged the responses to the two questions above (from -2, Liberal, to +2 Conservative). All continuous variables were scaled before entered into the analysis. We allowed the intercept to vary over subjects and argument contents. Table S2 summarizes the random and fixed effect of each model.

**Correlational study 1b**

*Participants.* In total, 605 participants (310 females, 295 males, Mean age = 45.5 years, SD = 16.8 years) took part in this study, who were recruited through MTurk, and completed the experiment online. In total, 357 people were Democrats, and 246 were Republicans.

Pre-registration of this study can be found at the OSF page of the project.

Materials and procedures are identical to Study 1a, with the exception of political knowledge scale which we did not administer to make the experiment shorter. The specifics of the statistical models are described in Table S2.

**Table S2**. Table shows the specifications of each model we run in Study 1a and 1b.

|  | Fixed | Random slope over Subjects | Random slope over item contents |
| --- | --- | --- | --- |
| Study 1a (MTurk) | Argument type, Partisanship | Argument type | Argument type |
|  | Argument type, CRT score,  Partisanship | Argument type | Argument type |
|  | Argument type, CRT score,  Partisanship,  Belief | Argument type | Argument type |
| Study 2a (Lucid) | Argument type, Partisanship | Argument type | Argument type |
|  | Argument type, CRT score,  Partisanship | Argument type | CRT score |
|  | Argument type, CRT score,  Partisanship,  Belief | Argument type | CRT score |

**Results**

In a first experiment, we used two sources of American participants, MTurk (Study 1a; N=300) and Lucid (Study 1b; N = 605 quota-matched to the US on age, gender, ethnicity, and geographic region). The two samples were analysed separately. The pre-registration for Study 1b can be found at: https://osf.io/fqkne/

In Study 1a, we first tested whether political preference was associated with how people judge pro versus contra climate change arguments (Figure S1). As expected, when predicting agreement we found a significant interaction between political partisanship and whether arguments were pro versus contra (argument type), b = 30.42, p < 0.0001; Republicans were more likely to agree with contra arguments than Democrats, while it was the other way around with pro arguments. This shows that there are indeed partisan differences in the evaluation of arguments pro and contra climate change; hence, this item set can be used to test the competing hypotheses.

| **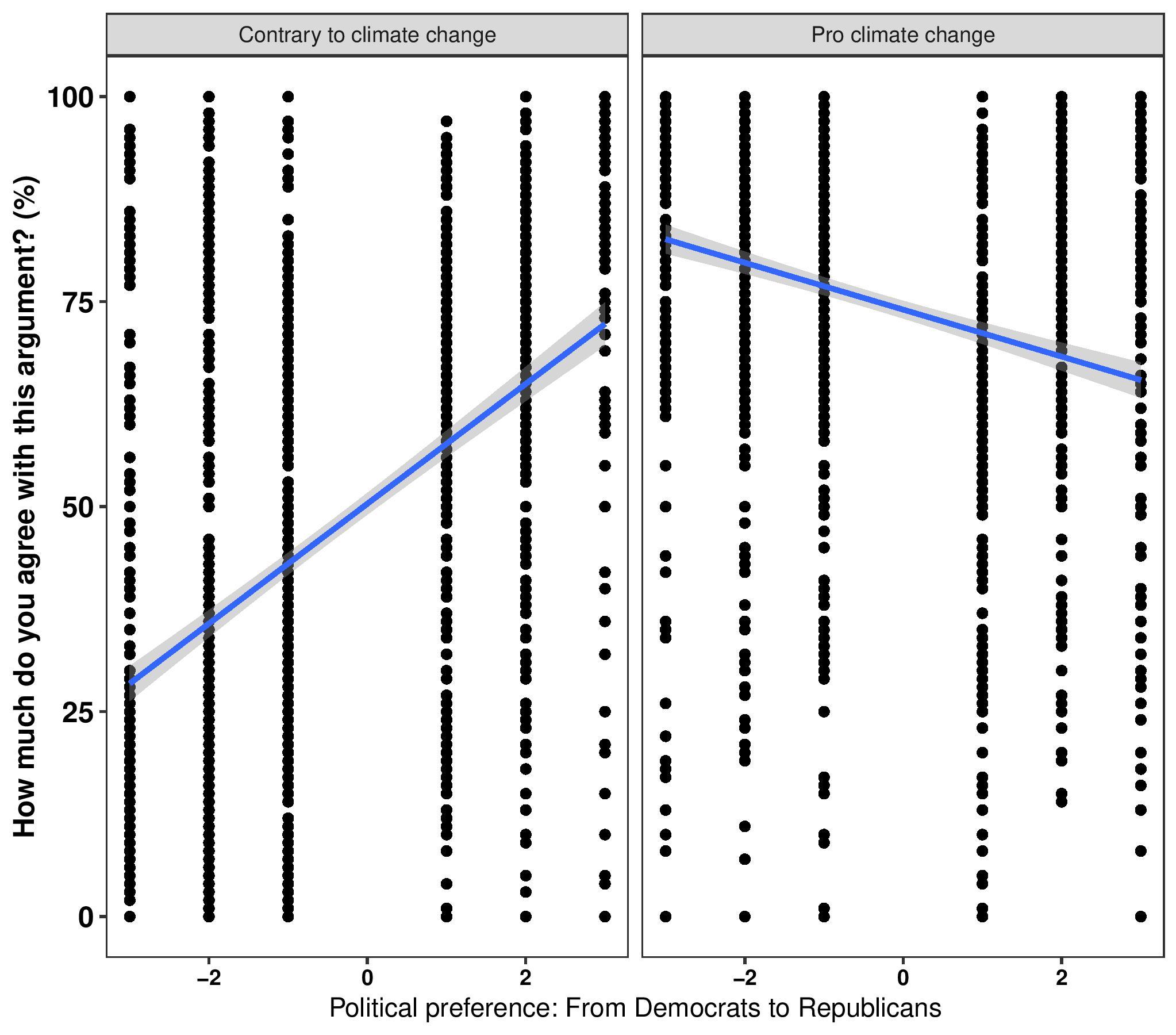** | **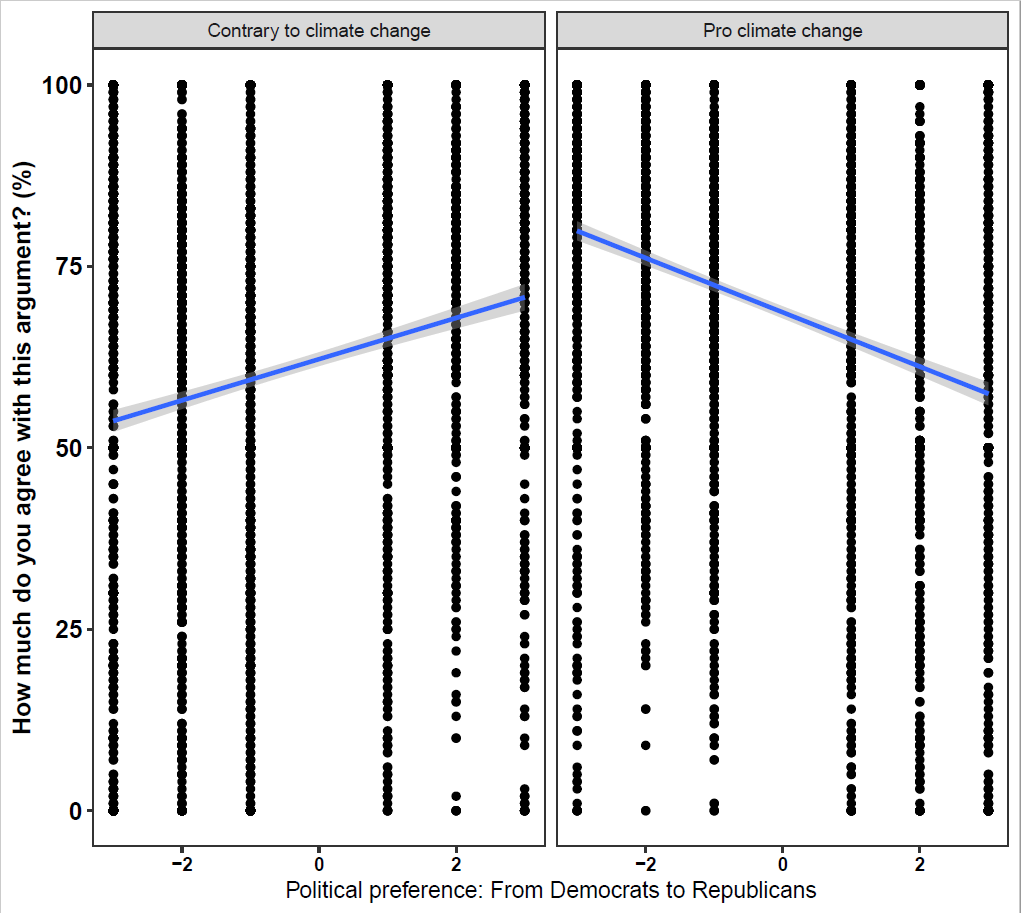** |
| --- | --- |

***Figure S1.*** *Agreement scores as a function of political partisanship and argument type on Mturk (left) and Lucid sample (right). Points represent trials.*

When adding cognitive sophistication score to the model we found a significant 3-way interaction between cognitive sophistication, argument type, and political partisanship, b = 32.59, p < 0.0001 (Figure S2). That is, the already existing partisan differences in argument evaluation were magnified by cognitive sophistication, providing a conceptual replication of Kahan et al (2011). Most importantly, however, when we added prior beliefs to the model, the 3-way interaction between political partisanship, argument type and cognitive sophistication was no longer significant, b = -6.6, p = 0.39; whereas there was a significant 3-way interaction between prior beliefs, argument type, and cognitive sophistication, b = 51.68, p < 0.0001. This suggests that cognitive sophistication magnifies the effect of prior beliefs rather than partisanship per se (Figure S3). Importantly, the correlation between beliefs about climate change and political partisanship (using a continuous measure of Democrat-Republican partisanship strength) was positive, but modest in size (r = 0.29), demonstrating that they are clearly separate (albeit related) measures (see Tappin et al., 2020 for further discussion).

Study 1b replicated these results using a larger and more representative sample: Democrats were more likely to agree with pro than with contra arguments, and it was the other way around for Republicans (i.e., significant interaction between argument type and political partisanship, b = 19.63, p < 0.0001); and while cognitive sophistication increased partisan differences when modeled without priors (i.e., significant three-way interaction between partisanship, cognitive sophistication and argument type), b = 53.12, p < 0.0001 (Figure S2), including prior beliefs in the model eliminated this interaction, b = 3.4, p = 0.34 but themselves showed a strong interaction with argument type and crt score, b = 38.5, p < 0.0001 (Figure 3).

| 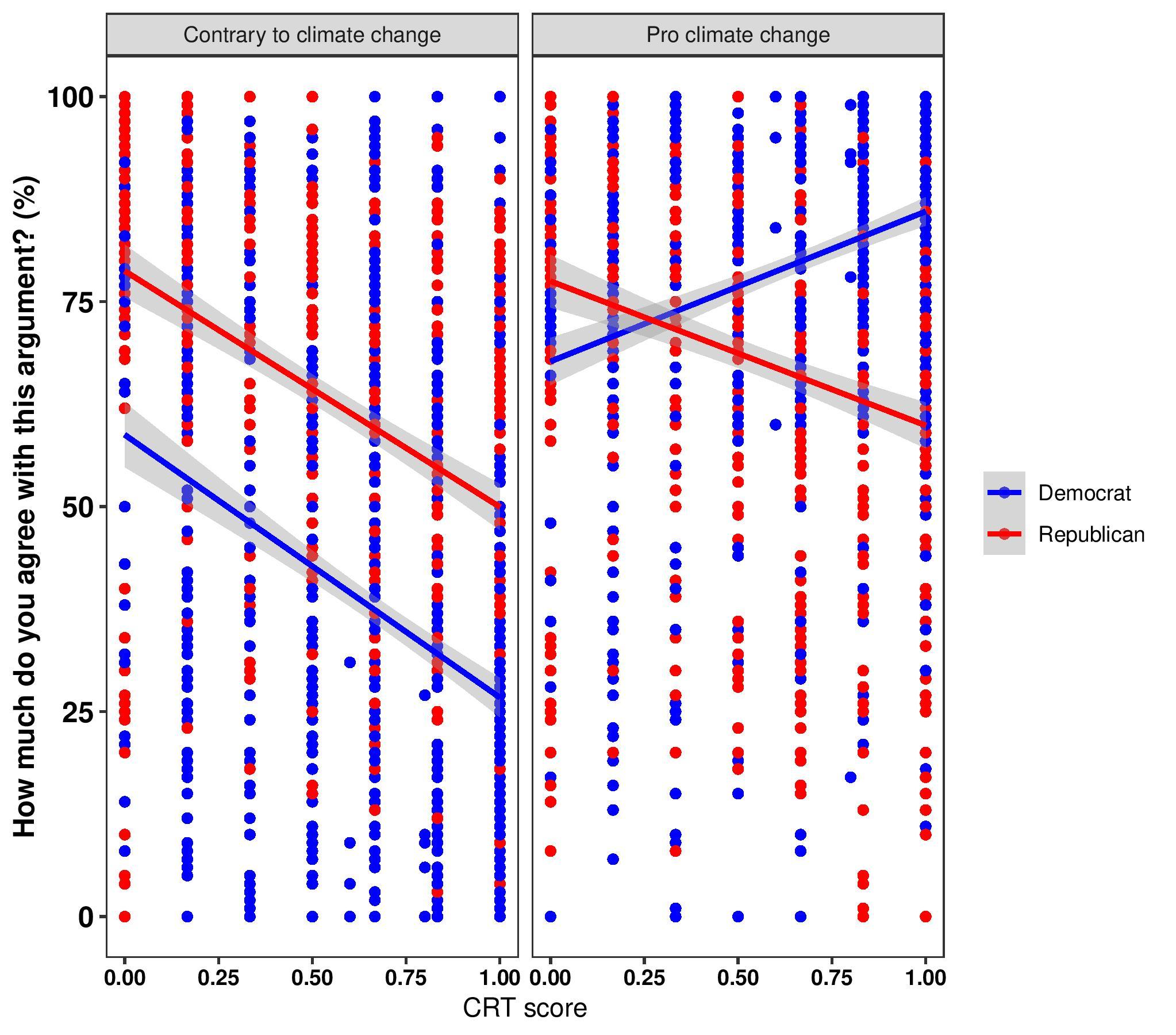 | **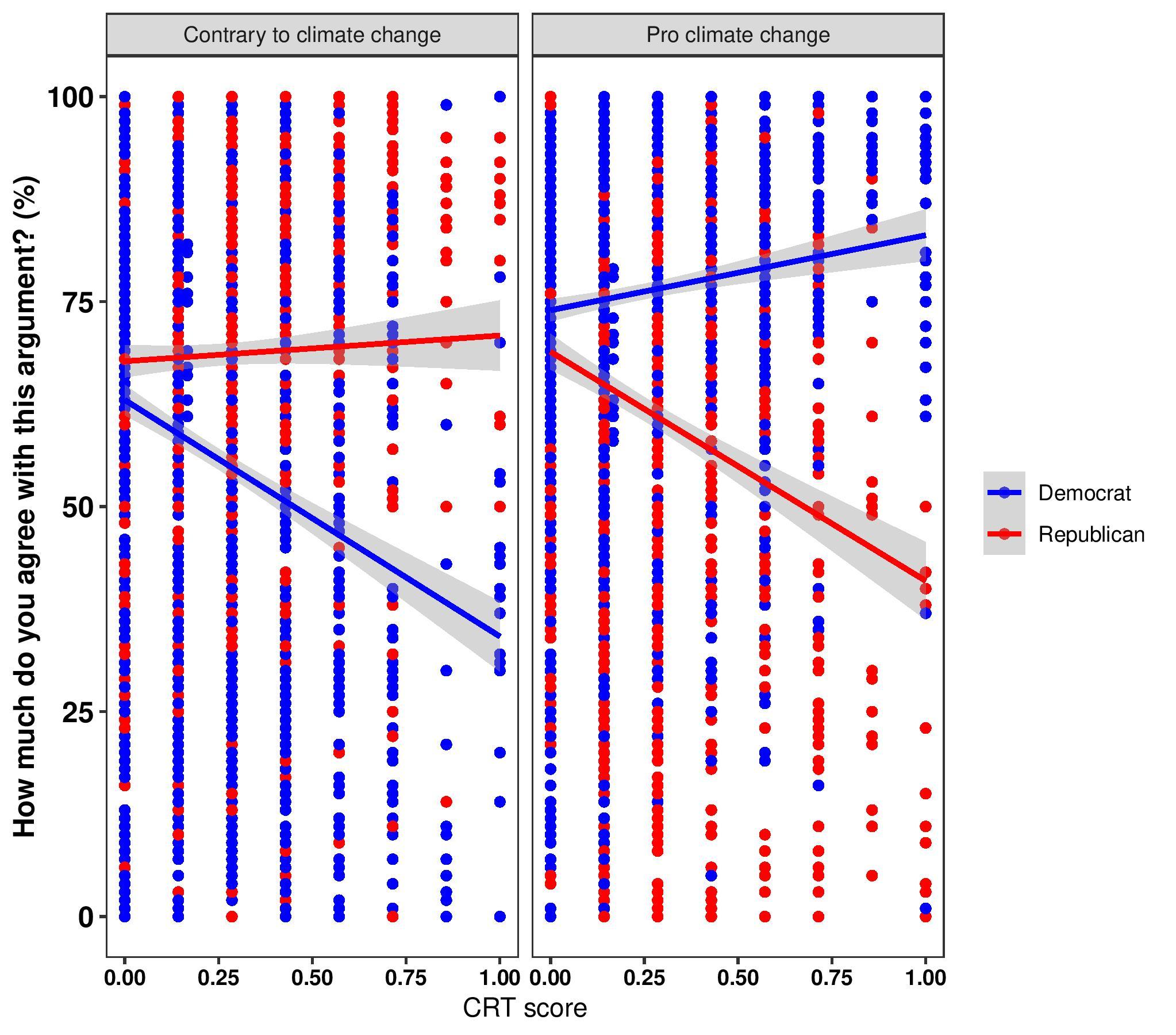** |
| --- | --- |

**Figure S2.** *Agreement scores as a function of political partisanship, CRT score and argument type on Mturk (left) and Lucid sample (right). Points represent trials.*

| **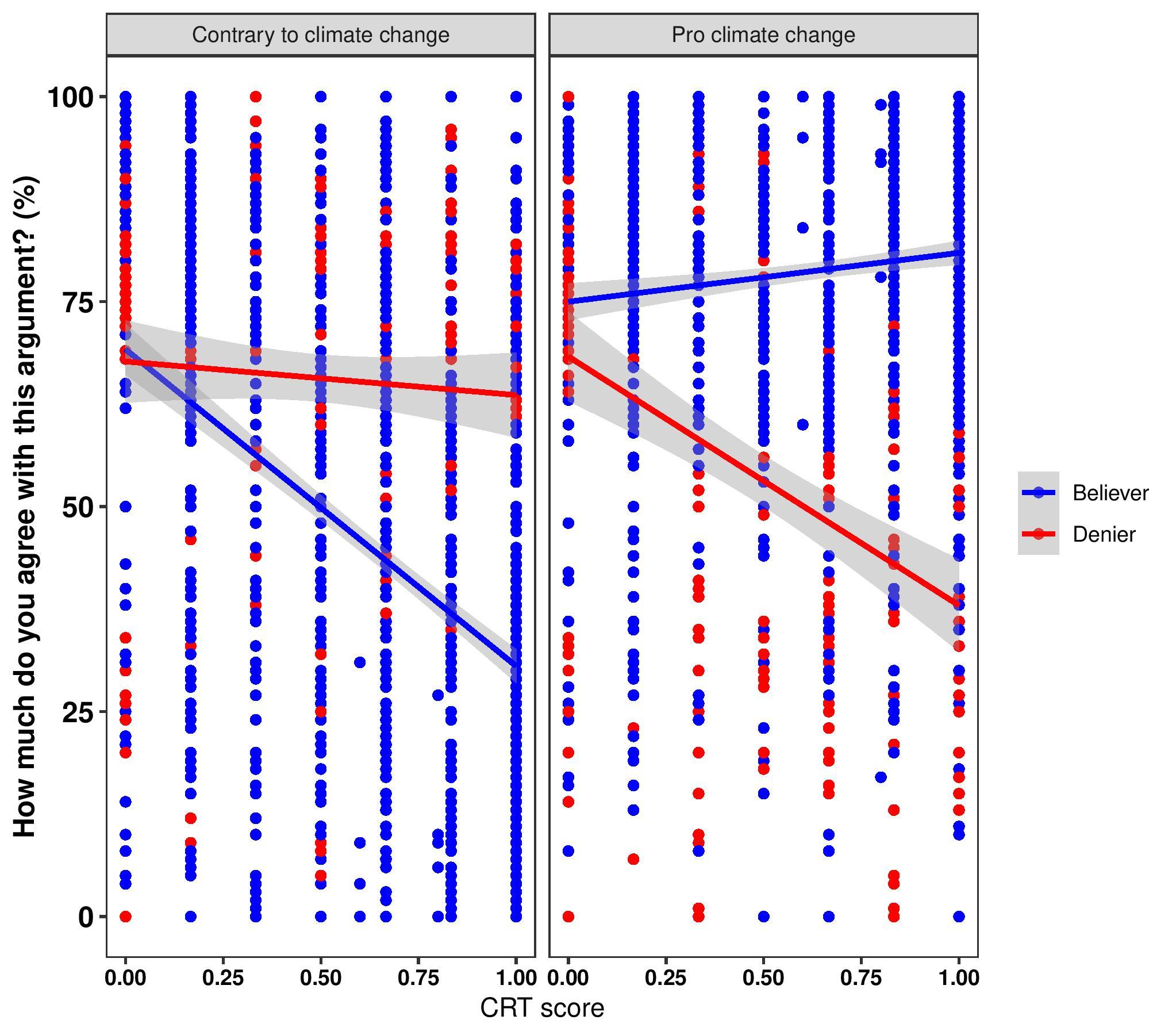** | 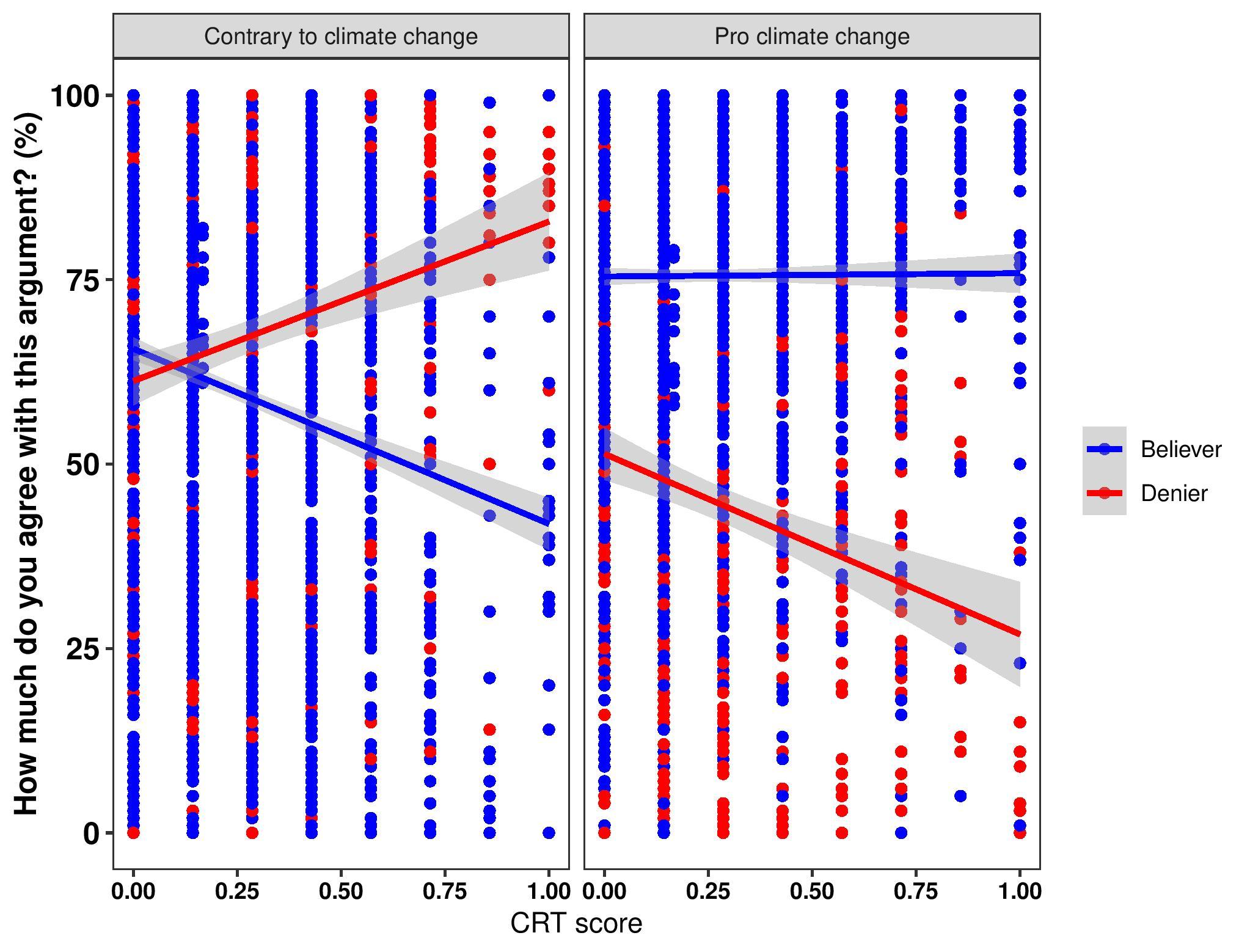 |
| --- | --- |

**Figure S3.** *Agreement scores as a function of prior beliefs, CRT score and argument type on Mturk (left) and Lucid (right) sample. Points represent trials.*

Additionally, we also conducted an analysis replacing partisanship with ideology (ideology was only recorded in Study 1a and not 1b). We found qualitatively equivalent results. There is a significant three way interaction between prior beliefs, argument type and CRT (measure of cognitive reflection abilities, see Supplementary Materials), b = 53.79, p < 0.0001, while there is no interaction between ideology, argument type and CRT, b = 1.97, p = 0.82. Hence, this supports our conclusion that it is prior beliefs that drive reasoning directly and not ideology or partisanship.

1. **Additional Results to the main, two response experiments.**

**Results**

**Table S3.** Table shows all the fixed and random effects that were included in the models in both studies. In all models, the intercept was allowed to vary over subjects and item contents.

|  | Fixed | Random slope over Subjects | Random slope over item contents |
| --- | --- | --- | --- |
| Study 1 | Argument type,  Response type (0 - final response, 1 - one response baseline),  Partisanship, Belief | Argument type | Argument type, Belief |
|  | Argument type,  Response type (0 - intuitive response, 1 - final response),  Partisanship, Belief | Argument type | Argument type, Belief |
|  | Argument type, Response type | Argument type | Argument type |
| Study 2 | Argument type,  Response type (0 - final response, 1 - one response baseline),  Partisanship, Belief | Argument type | Argument type, Belief |
|  | Argument type,  Response type (0 - intuitive response, 1 - final response),  Partisanship, Belief | Argument type | Argument type, Belief |
|  | Argument type, Response type | Argument type | - |
| Pooled | Argument type,  Response type (0 - intuitive response, 1 - final response),  Partisanship, Belief | Argument type | Argument type, Belief |

**Anchoring effect**

We compared the final response of people in the two response paradigm to the responses of people in the baseline one response condition (between subject comparison) to see if the final response stage is affected by anchoring effect (that is, people anchored to their initial response in the two response paradigm, and gave a final response having that in mind, presumably, to appear consistent). We see largely similar results across both studies. First, we found a significant two way interaction between argument type and response type in Study 1, but not in Study 2 (no other fixed effects included in the model, Study 1: b = 4, p = 0.033, Study 2: b = 3.3, p = 0.11). We found a significant three way interaction between response type (final response vs one response baseline), argument type, and belief (Study 1: b = 10.9, p = 0.002, Study 2 = b = 11.1, p = 0.01), while no significant interaction between response type, argument type and partisanship (Study 1: b = -6.3, p = 0.083, Study 2: b = -3.4, p = 0.491). Most importantly, the significant three way interaction suggests that people in the two response paradigm were indeed anchored to their initial response, rendering the within-subject test of the effect of deliberation on agreement scores less sensitive.

**Initial vs final response (within-subject deliberation effect)**

Although we found evidence for anchoring effect in the final response stage, for completeness, we compared the initial and final response stage of the two response paradigm (within subject comparison). We found a significant interaction effect between argument type and response type in both studies (no other fixed effects included in the model, Study 1: b = -1.6, p = 0.016, Study 2: b = -1.8, p = 0.028). After adding partisanship and prior belief into the model, we found different results in the two studies. In Study 1, we found no significant interaction between response type (initial vs final response) and argument type, b = 1.2, p = 0.27, also no interaction between argument type, response type and partisanship, b = -0.93, p = 0.587, but we found a significant interaction between argument type, response type and prior belief, b = -4.6, p = 0.003. In Study 2, but we did find a significant interaction between argument type, response type and partisanship, b = -4.7, p = 0.029, and also between response type, argument type and prior belief, b =-3.9, p = 0.04.

Since the two analyses had conflicting results (in study 1 we found no significant interaction with partisanship, while we did in study 2), we pooled the two samples together, and conducted the same analysis again. In this pooled analysis, we found no significant interaction between argument type, response type and partisanship, b = -2.4, p = 0.07, but the interaction between response type, argument type and prior belief was significant, b = -4.4, p = 0.0003. This overall supports our main conclusion that deliberation is driven by belief-coherence effect.

1. **Additional plots**

| 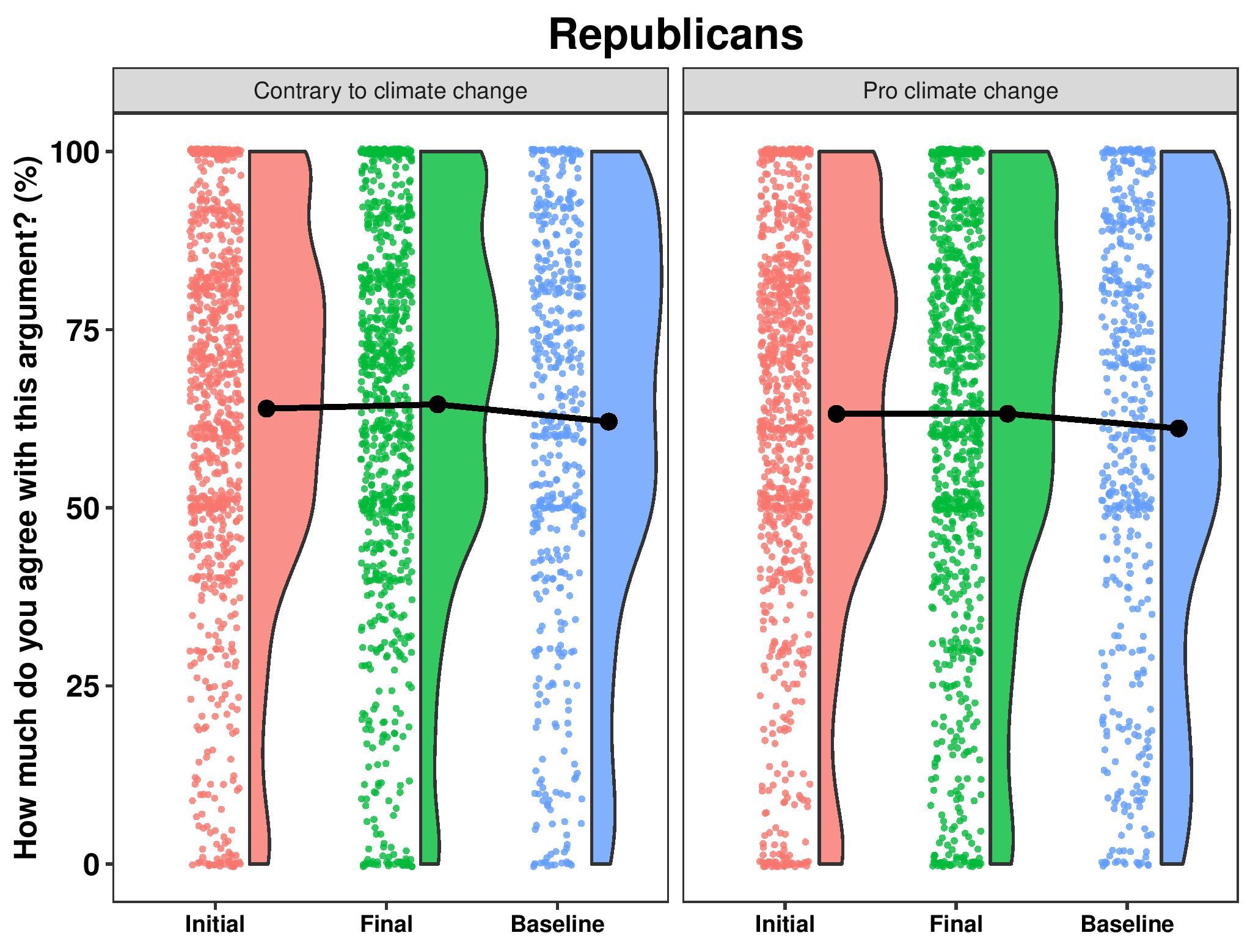 | 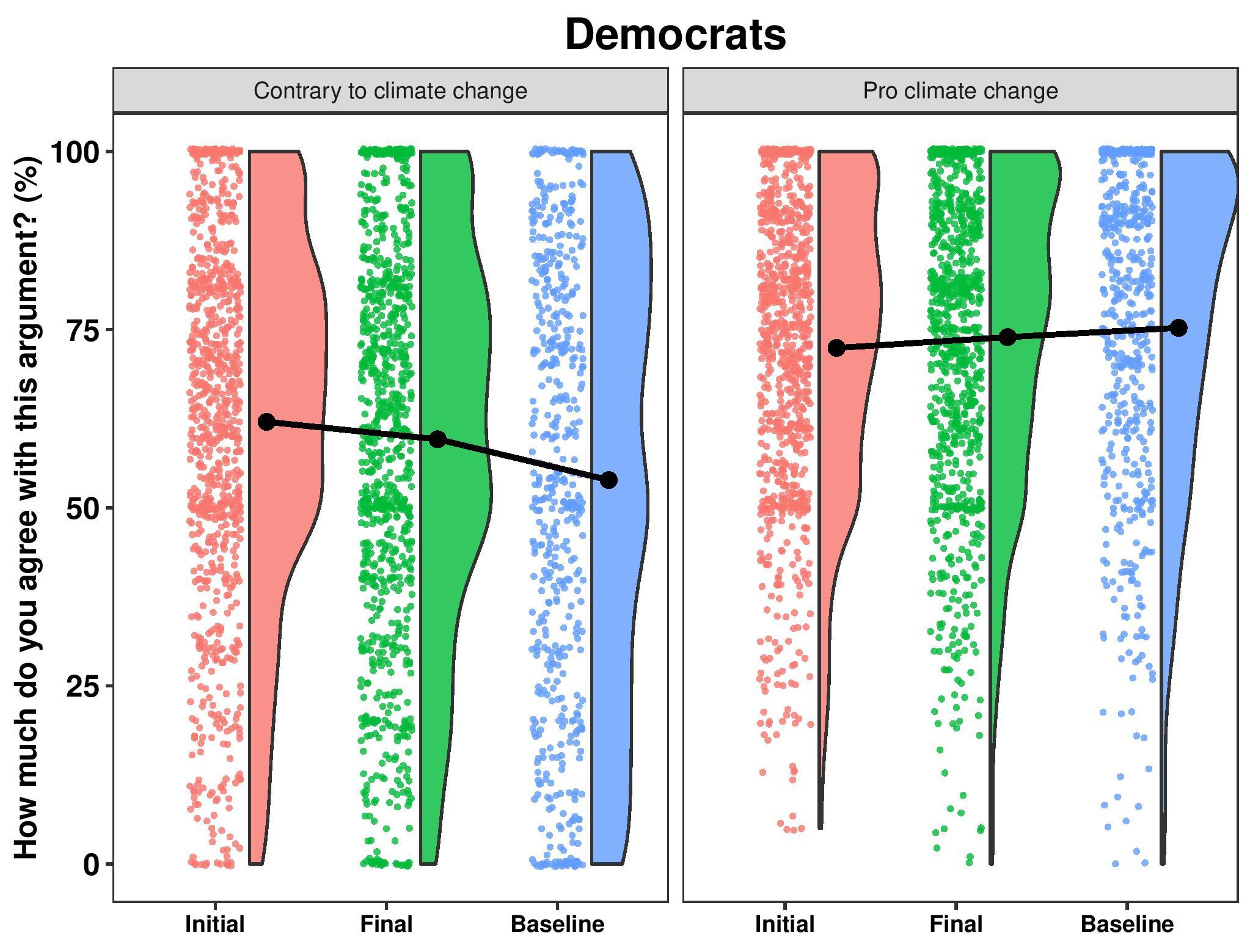 |
| --- | --- |

**Figure S4.** *Agreement scorers as a function of party affiliation, response condition (intuitive response = initial; deliberative response = final response (within-subject analysis), one-response baseline (between-subject analysis)), and argument type (Study 1). Dots in the middle of the half-violin plots are averages. Positive trends mean increase in belief after deliberation, while negative trends mean a decrease in belief after deliberation.*

| 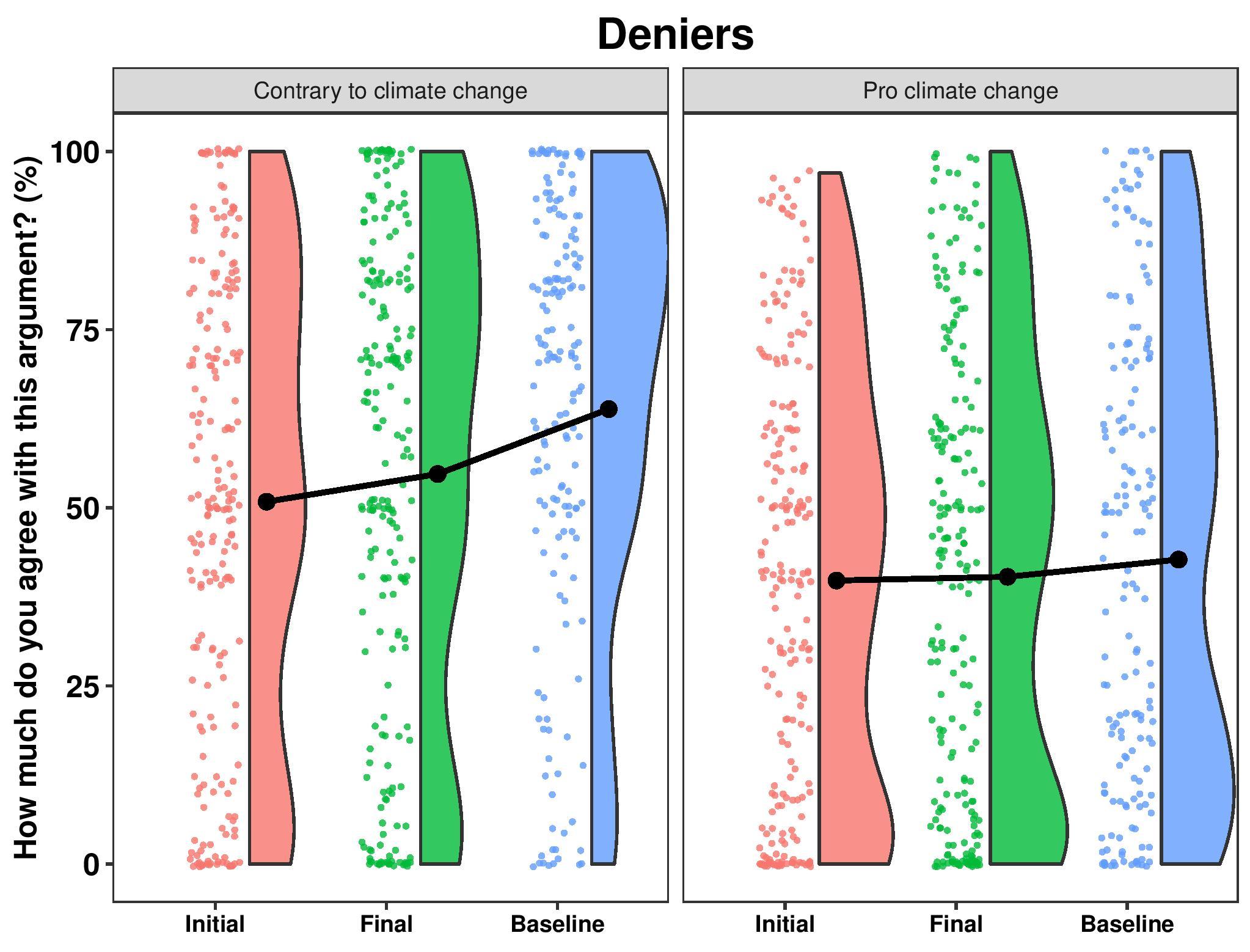 | 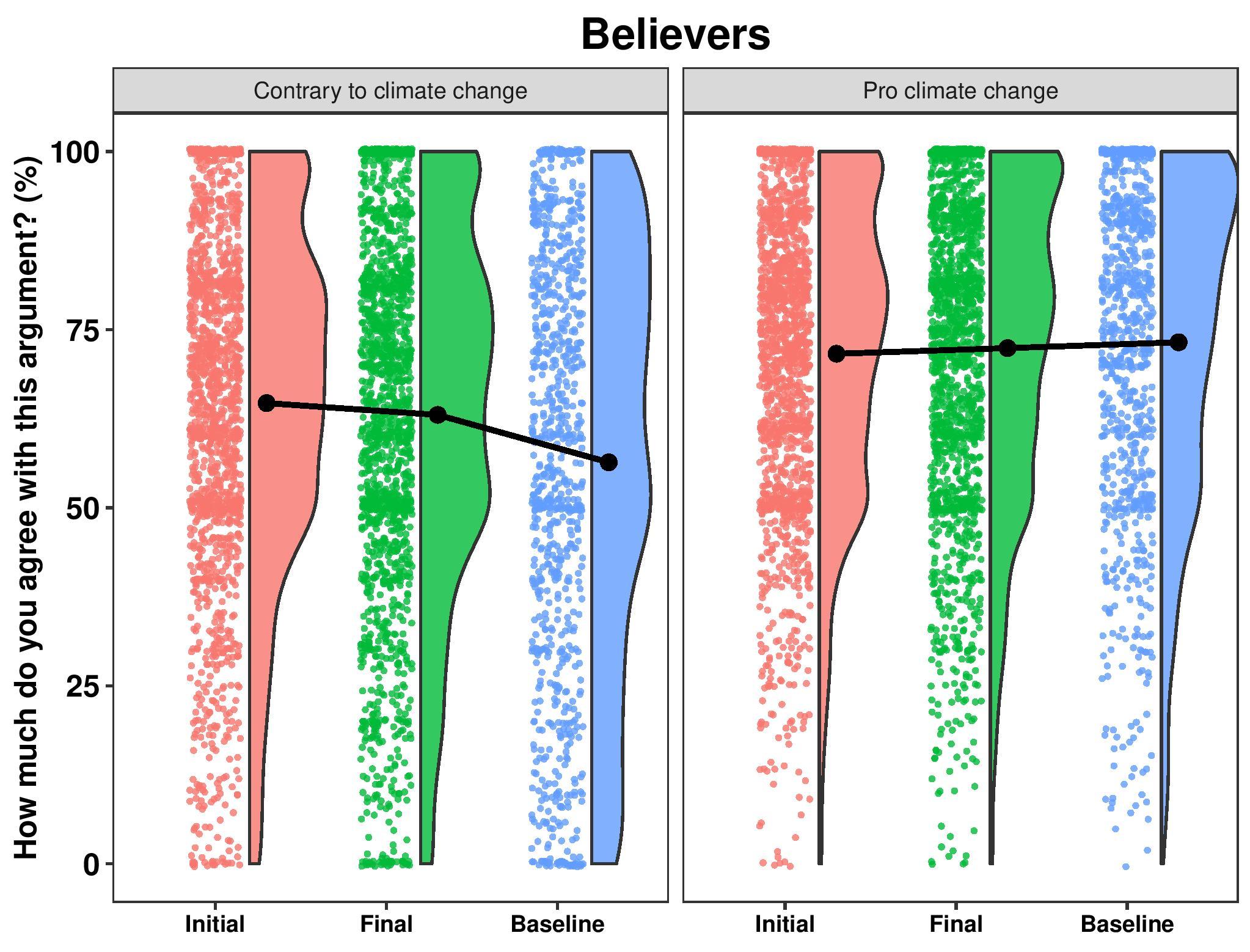 |
| --- | --- |

**Figure S5.** *Agreement scores as a function of prior beliefs, response condition (intuitive response = initial; deliberative response = final response (within-subject analysis), one-response baseline (between-subject analysis)), and argument type (Study 1). Dots in the middle of the half-violin plots are averages. Positive trends mean increase in belief after deliberation, while negative trends mean a decrease in belief after deliberation.*

| 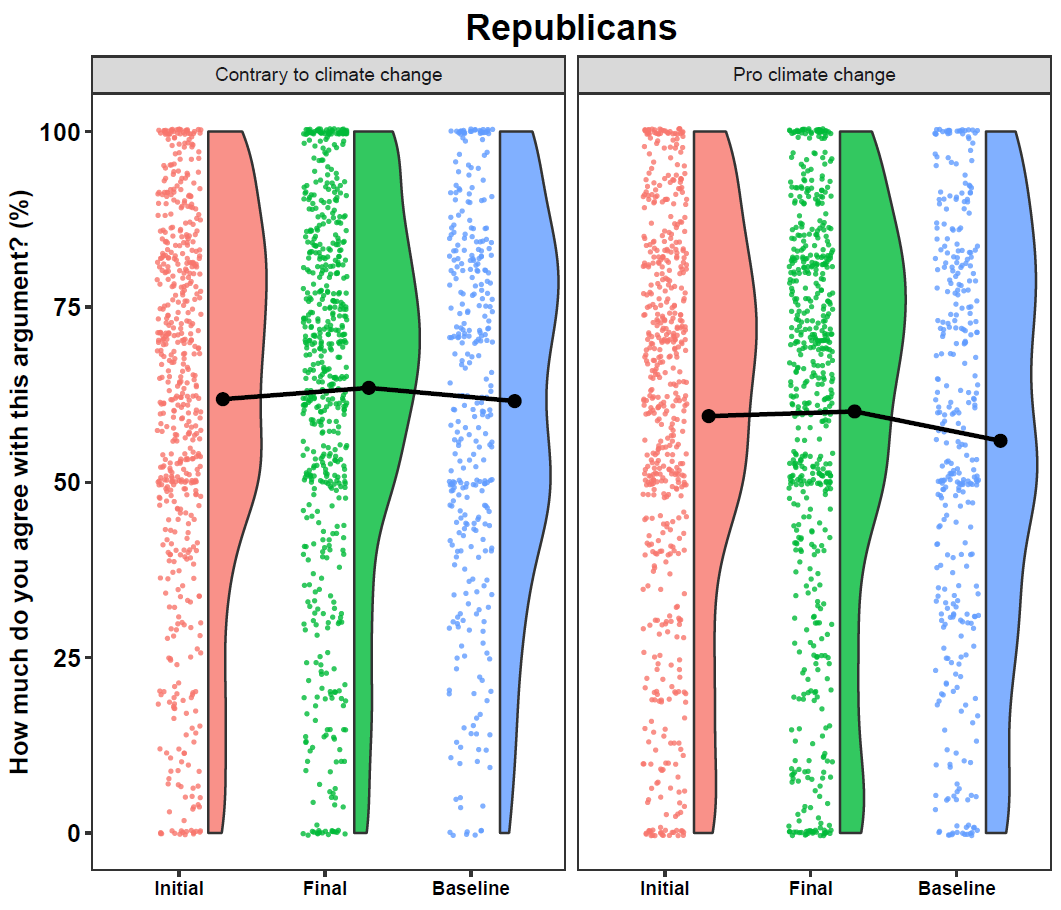 | 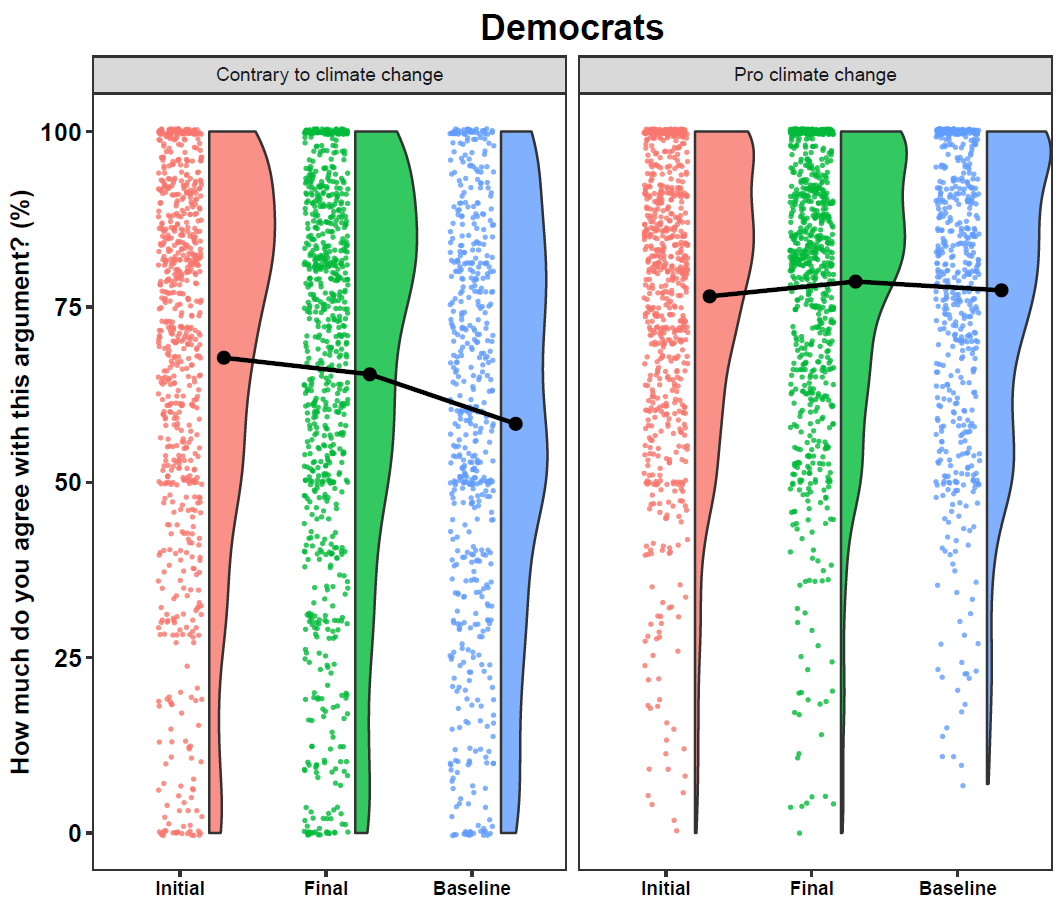 |
| --- | --- |

**Figure S6.** *Agreement scorers as a function of party affiliation, response condition (intuitive response = initial; deliberative response = final response (within-subject analysis), one-response baseline (between-subject analysis)), and argument type (Study 2). Dots in the middle of the half-violin plots are averages. Positive trends mean increase in belief after deliberation, while negative trends mean a decrease in belief after deliberation.*

| 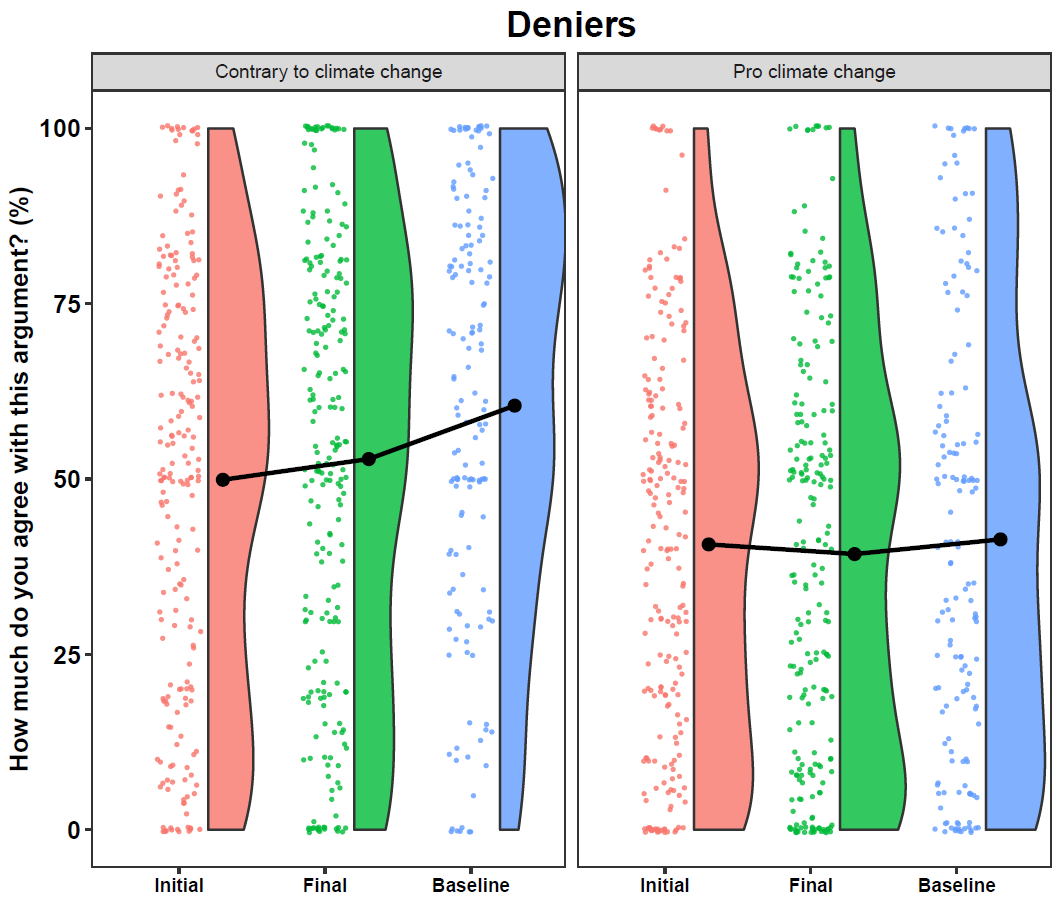 | 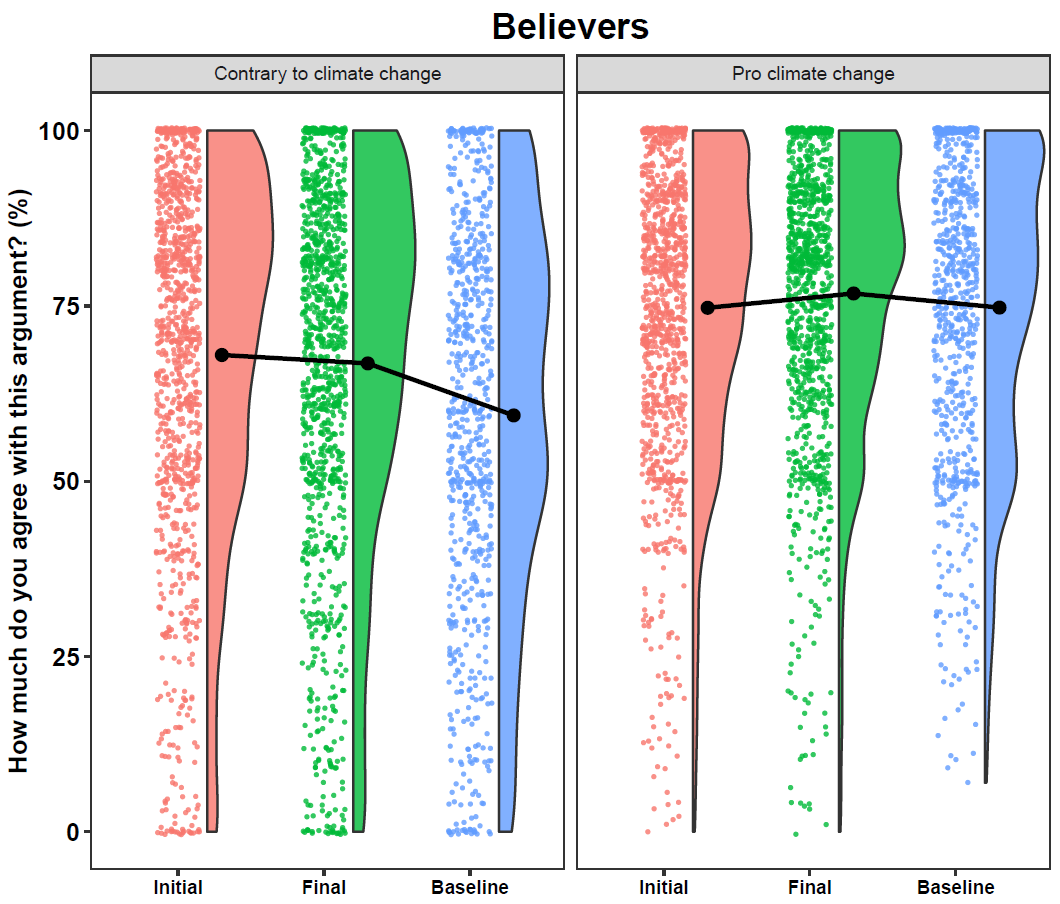 |
| --- | --- |

**Figure S7.** *Agreement scores as a function of prior beliefs, response condition (intuitive response = initial; deliberative response = final response (within-subject analysis), one-response baseline (between-subject analysis)), and argument type (Study 2). Dots in the middle of the half-violin plots are averages. Positive trends mean increase in belief after deliberation, while negative trends mean a decrease in belief after deliberation.*

*
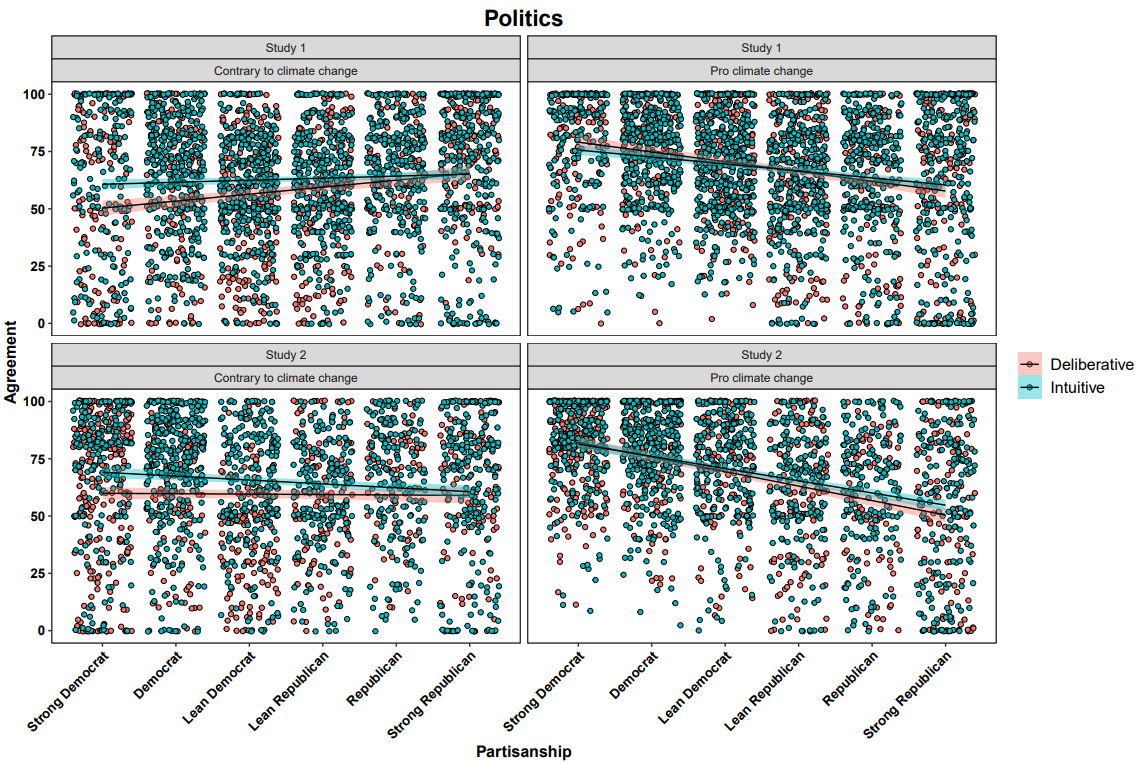
*

**Figure S8.** *Agreement scores as a function of partisanship, study, argument type and response condition (Deliberative = one response baseline, between-subject, Intuitive = Initial response, two response condition). Lines are regression lines.*


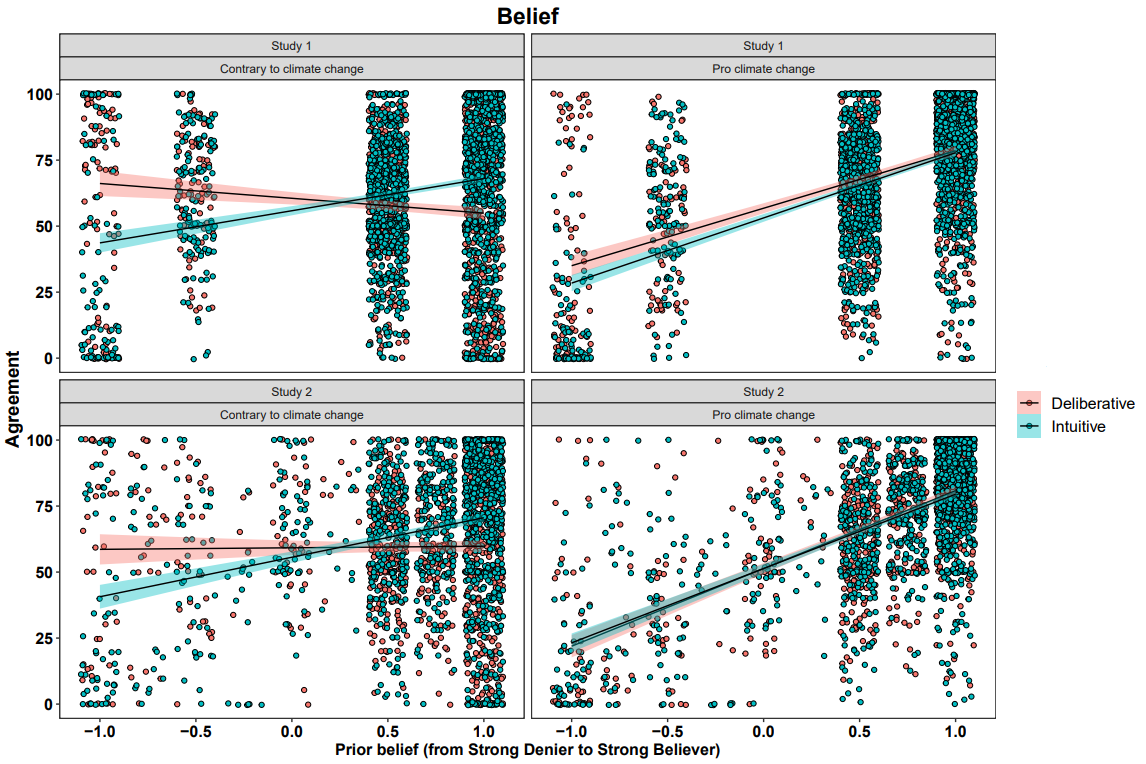


**Figure S9.** *Agreement scores as a function of prior belief, study, argument type and response condition (Deliberative = one response baseline, between-subject, Intuitive = Initial response, two response condition). Lines are regression lines.*

1. **Standardized regression coefficients for Study 1 and 2, Intuitive vs Deliberative (initial response vs one response baseline) comparison.**

**Table S4.** Standardized regression coefficients for Study 1 and 2.

|  | Study 1 | | Study 2 | |
| --- | --- | --- | --- | --- |
|  | Missed trials ignored | Missed trials replaced | Missed trials ignored | Missed trials replaced |
| Argument type * response type | *b* = -0.21  *p* = 0.001 | *b* = -0.21  *p* = 0.001 | *b* = -0.19  *p* = 0.005 | *b* = -0.18  *p* = 0.007 |
| Politics * argument type * response type | *b* = -0.05  *p* = 0.43 | *b* = -0.05  *p* = 0.39 | *b* = -0.04  *p* = 0.61 | *b* = -0.03  *p* = 0.66 |
| Belief * argument type * response type | *b* = -0.34  *p* < 0.0001 | *b* = -0.33  *p* < 0.0001 | *b* = -0.28  *p* = 0.002 | *b* = -0.29  *p* = 0.001 |
| Deniers - Contra arguments: Response type effect | *b* = 0.41  *p* = 0.008 | *b* = 0.41  *p* = 0.009 | *b* = 0.33  *p* = 0.03 | *b* = 0.38  *p* = 0.01 |
| Believers - Contra arguments: Response type effect | *b* = -0.3  *p* < 0.0001 | *b* = -0.3  *p* < 0.0001 | *b* = -0.32  *p* < 0.0001 | *b* = -0.33  *p* < 0.0001 |
| Deniers - Pro arguments: Response type effect | *b* = 0.1  *p* = 0.53 | *b* = 0.09  *p* = 0.56 | *b* = 0.01  *p* = 0.95 | *b* = 0.02  *p* = 0.9 |
| Believers - Pro arguments: Response type effect | *b* = 0.07  *p* = 0.28 | *b* = 0.07  *p* = 0.27 | *b* = 0.003  *p* = 0.96 | *b* = -0.03  *p* = 0.69 |
| Republican Deniers - Pro arguments: response type effect | *b* = 0.43  *p* = 0.02 | *b* = 0.42  *p* = 0.02 | *b* = 0.44  *p* = 0.009 | *b* = 0.47  *p* = 0.005 |
| Republican Believers - Pro arguments: response type effect | *b* = -0.25  *p* = 0.009 | *b* = -0.25  *p* = 0.01 | *b* = -0.26  *p* = 0.03 | *b* = -0.28  *p* = 0.02 |

1. **Results using ideology rather than partisanship**

We tested the robustness of our results to using ideology rather than partisanship in our models (as well as, of course, prior beliefs, condition [initial or baseline] and argument type). Although we did not measure ideology in our experiments, we managed to recover an ideology measure (7 point Likert scale from Extremely liberal to Extremely conservative) for 582 participants (pooling across S1 and S2) from records maintained by Lucid, the subject recruitment platform used for the study. Analyzing these the responses of these 582 participants, we found no significant three way interaction between ideology, condition and argument type, b = -5.03, p = 0.41, while finding a significant three way interaction between prior beliefs, condition and argument type, b = -13.7, p = 0.01. These results, along with the correlational evidence from Study 1a reported above in Supplementary Materials Section B, show that the same pattern observed for partisanship also holds for ideology: neither partisanship nor ideology are associated with the effect of deliberation once prior beliefs are accounted for.

1. **Correlates of trial exclusion**

Trial exclusion analysis was done using mixed effect logistic regression models, including the random intercept of subjects. All terms were separately analysed due to convergence issues in the model that included all terms (that was potentially caused by the small N of missed trials).

**Table S5.** Correlates of trial exclusion (Missed trial: 1; Answered: 0) in the pooled subject data (the two studies analysed together) in the intuitive response condition. Betas are standardized coefficients.

|  | **b** |
| --- | --- |
| **Age** | 0.02** |
| **Gender (Male:0.5; Female: -0.5)** | -0.38* |
| **CRT score** | 0.005 |
| **Partisanship (-1 Republican; +1 Democrat)** | 0.17* |
| **Prior belief** | 0.08 |
| **Argument type (Pro: 0.5; Contra: -0.5)** | -0.02 |
| **Argument type*Belief** | -0.05 |
| **Argument type*Politics** | -0.04 |

**Note. *<0.05, **<0.001**

1. **Assessing order effects and fatigue**

To assess potential fatigue caused by the demanding experiment, we tested order effects on agreement scores, reaction times and accuracies on the dot matrix task (the cognitive load task) in the “intuitive” condition. We found no effect of presentation order on agreement scores (Study 1: b = -0.03, p = 0.85; Study 2: b = -0.03, p = 0.881), and no effect separately for pro (Study 1: b = 0.23, p = 0.269; Study 2: b = 0.36, p = 0.123) or contra arguments (Study 1: b = -0.21, p = 0.37; Study 2: b = -0.5, p = 0.08). However, we did find that participants are speeding up during the experiment (i.e., taking less time to respond on arguments they are presented with later (Study 1: b = -0.44, p < 0.0001; Study 2: b = -0.41, p < 0.0001), probably suggesting that participants are getting used to the demanding experiment. This is confirmed by the fact that there is no order effect on load accuracies, suggesting that participants are not getting less impatient or inattentive (Study 1: b = 0.021 p = 0.336; Study 2: b = 0.03, p = 0.261). In fact, the positive beta suggests that there is a slight trend that by the end of the experiment they are getting more dot matrix problems right; also supporting that improving reaction times simply mean that they are getting used to the hard task.
